# Community and health systems learning: Evaluation of the VAPAR ‘learning platform’ experience in Mpumalanga, South Africa 2017-23

**DOI:** 10.12688/wellcomeopenres.23381.1

**Published:** 2025-03-18

**Authors:** Sophie Witter, Lucia D'Ambruoso, Maria van der Merwe, Jennifer Hove, Nombuyiselo Nkalanga, Denny Mabetha, Gerhard Goosen, Jerry Sigudla, Stephen Tollman, Verbal Autopsy with Participatory Action Research (VAPAR)/Wits/Mpumalanga Department of Health Learning Platform

**Affiliations:** 1 Institute for Global Health and Development, Queen Margaret University, Edinburgh, Musselburgh, Scotland, UK; 2 Aberdeen Centre for Health Data Science, Institute of Applied Health Sciences, School of Medicine, Medical Sciences and Nutrition, University of Aberdeen, Aberdeen, Scotland, UK; 3 MRC/Wits Rural Public Health and Health Transitions Research Unit (Agincourt), School of Public Health, University of the Witwatersrand, Johannesburg, Gauteng, South Africa; 4 National Health Service (NHS) Grampian, Aberdeen, Grampian region, UK; 5 Department for Epidemiology and Global Health, Umeå University, Umeå, Västerbotten County, Sweden; 6 Department of Global Health, Stellenbosch University, Stellenbosch, Western Cape, South Africa; 7 MV Consulting, White River, South Africa; 8 Mpumalanga Department of Health, Mbombela, South Africa

**Keywords:** learning platform; community health; health system strengthening; learning health systems; South Africa; participatory action research; theory-based evaluation

## Abstract

**Background:**

Learning platforms support community and health systems evidence generation and uptake but are complex, and there are few published evaluations. We present a theory-based evaluation of a learning platform in the rapidly transitioning context of rural South Africa 2017-23. The platform, called VAPAR (Verbal Autopsy with Participatory Action Research), aimed to embed a system of knowledge production and exchange for health systems strengthening to improve services and outcomes for under-served groups.

**Methods:**

Following a realist-informed protocol, we combine data across five reflection and action cycles from internal and external sources, including project evaluations of each cycle, programme reports, endline interviews (n=22), external reports and secondary data. Data are analysed against a programme theory of change.

**Results:**

VAPAR has been contextually relevant, adaptive, and created spaces for respectful, inclusive dialogue between local stakeholders, despite complex hierarchies characterising local health systems and public health emergencies. There is evidence of growth in skills, confidence, motivation and agency, especially amongst community health workers, and greater clarity on their roles. Relevance of community voice has been raised, and capacity built to support community health research. Relationships have been developed or reinforced between levels of the health system, and across sectors such as education and social welfare. Some gains are documented in health seeking and service provision, with the potential for longer-term impacts on health outcomes and equity. There has also been considerable investment in sharing tools and lessons within the country, regionally and internationally.

**Conclusions:**

Developing, embedding, and sustaining a functioning learning platform at scale is ambitious and we highlight some of the tensions and trade-offs involved, including challenges with sustainability. However, the evaluation does support the original proposition that bringing empowered community voices into decentralised health systems planning and decision-making is both feasible and impactful, emerging through collective, pragmatic and adaptive processes.

## Plain language summary

This study evaluated the Verbal Autopsy with Participatory Action Research (VAPAR) learning platform in Mpumalanga province, South Africa. The platform was developed to strengthen health systems by involving communities in health decision-making. Conducted over the lifetime of the programme from 2017–23, the evaluation used a theory-based approach, collecting data from five reflection and action cycles to understand how local knowledge production and exchange had impacted on various domains, including working relationships, demand for evidence and improved health services. We find that VAPAR adapted to challenges, such as the COVID-19 pandemic, and fostered open dialogue among health workers and community members despite existing power dynamics. The platform enhanced the skills, confidence, and roles of community health workers (CHWs). CHWs gained new skills in community mobilization, analysis, and peer support, and their roles were better recognized by the health system and the community. This led to stronger trust between communities and authorities and more evidence-based decision-making. Despite successes, challenges included sustaining support and managing complex stakeholder relationships. Learning platforms like VAPAR can be effective but require sufficient resources and ongoing support for long-term impact and equitable healthcare. The study’s limitations include the difficulty of assessing such complex programmes and its reliance on qualitative data.

## Introduction

Efforts to create learning health systems that address complex challenges by involving stakeholders in sustained and reiterative cycles of reflection, evidence generation, action, and learning are gaining attention. However, there is little evaluation of strategies for building such systems in low- and middle-income countries (LMICs) ^1^. This paper contributes to this field by sharing lessons from a participatory evaluation of the VAPAR programme, implemented in Mpumalanga, South Africa, 2017–23. The findings offer practical insights into strengthening community-health sector collaborative approaches to health systems improvement through deeper, multi-level cooperation—an area acknowledged as a critical gap in health systems research ^2^.

VAPAR (Verbal Autopsy with Participatory Action Research) is based in Mpumalanga, South Africa, a rural province of 5.14 million people in the northeast ^i^. In poor and rural villages there is limited piped water, rudimentary sanitation, underdeveloped roads, unaffordable electricity and high unemployment ^3^. The HIV burden is high and highly unequal. Nationally, prevalence in black populations is 40–50 times that of white and in adolescents, risks are 8 times higher in female adolescents than males ^4^. HIV prevalence in the study area was 26% in women and 19% in men ^5^. National data for 2022 found that Mpumalanga province had the highest HIV prevalence in the country at 17.4% ^ii^.

South Africa’s post-apartheid health and social policies are widely regarded as progressive and inclusive ^6^, reflecting a constitutional commitment to the right to health and community participation in primary health care (PHC) ^7^. The introduction of National Health Insurance (NHI) in 2012 further underscored the country’s ambition for Universal Health Coverage (UHC) ^8^. However, promising policy frameworks co-exist alongside significant challenges. Chronic underfunding of public services, an entrenched culture of accountability directed upward rather than toward communities, persistent workforce shortages, corruption, poor governance, and failing infrastructure have created a critical gap between policy intentions, implementation capabilities, and outcomes ^9^. Additionally, the health system is burdened by a complex interplay of health challenges, including high rates of chronic infectious diseases such as HIV/AIDS and tuberculosis, non-communicable diseases, maternal and child mortality, and deaths resulting from violence and injuries ^10^.

The VAPAR programme started in 2017 as a partnership of local and international researchers, community members and health system stakeholders. Its aim was to embed a system of knowledge production and exchange for health systems strengthening to improve health services and outcomes for vulnerable groups locally and with the potential, if successful, for wider learning, uptake and sustainability ^iii^. The collaboration was based in Bushbuckridge sub-district, then with a population of 550,000. Within the sub-district, the programme was hosted by a Health and Socio-Demographic Surveillance System (HDSS), the MRC/Wits Rural Public Health and Health Transitions Unit (Agincourt). Wits/Agincourt is among Southern Africa’s oldest and largest population-based cohorts, covering a defined population of approximately 130,000 living in 31 villages over 450km ^2^ ^3^.

In VAPAR, data from Verbal Autopsy (VA) and Participatory Action Research (PAR) are combined through reiterative action/reflection cycles aiming at continuous quality improvement for health systems strengthening, and engaging relevant stakeholders at different levels, including rural communities. The programme was intended to consist of three learning-and-action cycles 2017–23, with each cycle of the VAPAR programme comprising defined stages: engage/observe; analyse/plan; and act/reflect ^1^. 

The VA component incorporated new World Health Organization (WHO) indicators developed with the Mpumalanga Provincial Department of Health (DoH) during pilot work 2015-16 which informed, in the main phase, a novel classification system contributing social and health system circumstances within VA data gathered through the MRC/Wits Agincourt HDSS. Data on local levels, causes and circumstances of deaths (based on VA outputs) were shared during PAR with village-based groups, and in which priority health topics were identified. The process analysed root causes and impacts of the problem, identified stakeholders and planned collective action to address these.

In the pilot phase 2015–16, substantive topics were investigated including under-5 mortality, HIV and violence based on burden of disease analysis ^6, 11, 12^. In the main programme, communities nominated focus topics in the first cycle, and thereafter, communities and health systems stakeholders collectively nominated. During the evaluation period 2017–23, focus topics were: access to clean water, alcohol and drugs (Cycles 1-2/community priorities), and HIV/TB treatment (Cycles 3-5/shared priority) .

Statistical data on levels, causes, and circumstances of deaths (from VA), combined with qualitative evidence on lived experience (from PAR), and new, peer learning capabilities (among community partners) were the basis for collective learning with non-peer stakeholders. Non-peer stakeholders included health planners, managers and workers from different levels and sections, and from adjacent sectors and parastatals as relevant. Through regular dialogue, new data and evidence were appraised in learning spaces facilitated by researchers. The process built common understandings of problems and root causes. From this, local actionable agendas were collectively developed, implemented and evaluated. Democratic and participatory principles promoted learning and action in processes that focussed on deepening relationships and trust (‘software’), together with embedding learning spaces and processes in the system (‘hardware’).

## Methods

This section describes the approach and evaluation methods, full details are contained in the evaluation protocol ^1^. Reflecting the VAPAR approach, the evaluation was participatory, building on reflections and insights of partners and wider stakeholders generated in the PAR cycles, and focussed on joint learning on whether and how VAPAR contributed to its goals, and what could be learned for this and other settings. Participatory evaluation is a growing field ^13^. This evaluation had unique benefits and challenges evaluating an intervention which was itself participatory, multi-level, multi-cycle, pragmatic, emergent and embedded in a rapidly changing context ^1^.

### Theory of change development

**Figure 1.**
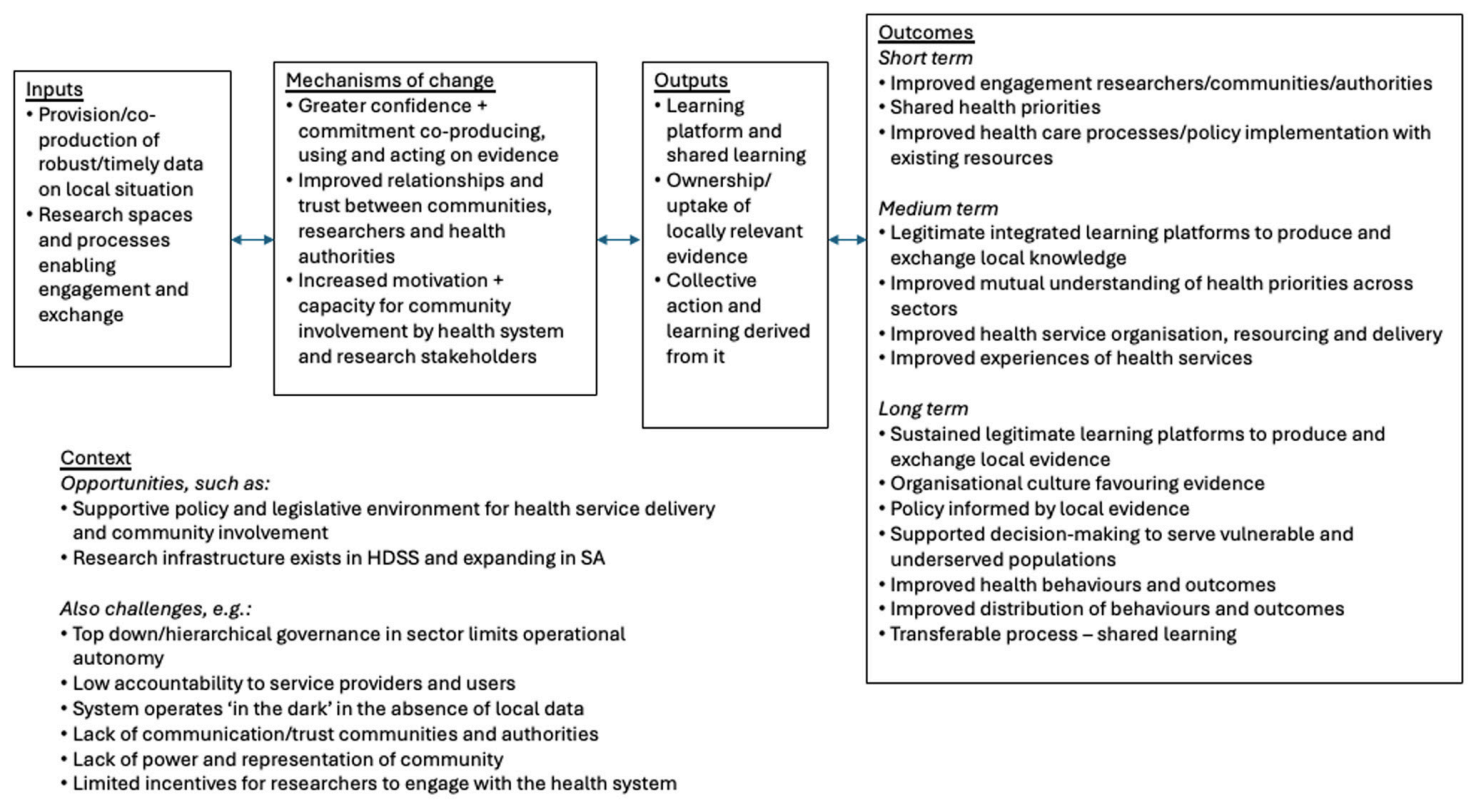
Initial VAPAR theory of change. (This figure has been reproduced with permission from Source: 15 under a Creative Commons Attribution 4.0 International License, which permits use, sharing, adaptation, distribution and reproduction in any medium or format, http://creativecommons.org/licenses/by/4.0/).

A realist-informed theory of change was developed by the research team to reflect on the programme and to structure the evaluation. During the first VAPAR cycle 2017–19, the research team developed an initial theory of change, based on continuous interactions with community, health system and public administration stakeholders, as well as wider literature and data analyses ( Figure 1). The theory of change considers the challenges and resources in context, the expected causal pathways, including change mechanisms and their underpinning assumptions, and desired outcomes, following a realist schema. While presented in a linear fashion, the stages are connected, fluid and in continuous interaction, with mechanisms key to bringing about change. The learning cycles present opportunities to engage in and analyse repeated interactions over time. In each cycle, data were collected to refine our understanding of the intervention. This included qualitative and quantitative data collected by the programme (on context, inputs, activities, outputs, outcomes and assumptions), supplemented by end-of-cycle reflexive evaluation interviews and workshops with stakeholders from community to provincial levels. These were reviewed by the programme team, key stakeholders, and an independent advisory panel, leading to refined engagement and an updated theory of change at the end of each cycle. Here, we assess VAPAR using the original theory of change ( Figure 1).

### Sources

The evaluation draws on a variety of perspectives and data, including programme data and secondary data analyses. This was supplemented by key informant (KI) in-depth interviews and workshops at the end of each cycle to probe the different domains, understand changes to the positions of different actors within the local health system, and feed back into improved learning and action in the next cycle. Pre/post interviews with CHWs were conducted for each of cycles 3-5 in addition to the reflective workshops with wider stakeholders. For cycle 3 this included all CHWs trained ^14^. For cycles 4 and 5, a sample was drawn. Where these have been previously analysed and published, we refer to the published paper for details on methods, rather than re-describing them here. Quantitative data such as from VA were analysed for significant trends in health indicators for different population groups. However, the bulk of the data was qualitative, reflecting the focus of the programme on changing ‘software’ such as relationships, trust, attitudes and skills. Data sources are summarised in Table 1, below. For details on how sources map to the domains of the theory of change, see 1.

**Table 1.** Summary of data sources for evaluation (Source: authors)

Data sources	Description
*Internal to VAPAR*	
VA and PAR outputs	e.g. analyses of VA data, Photovoice images, root cause mapping using problem tree, Venn diagrams, action pathways produced by community participants across five VAPAR cycles
Cycle reflections and evaluations	Published for cycles 1, 2 and 3; and evaluation papers on cycles 4 and 5 are forthcoming (reports on these cycles are used instead in this evaluation)
Published VAPAR research papers	25 published to date; these are referenced on relevant topics
Evidence briefs	13 produced in English and Tsonga, feeding evidence on chosen priority topics to community and system stakeholders
Local action plans and follow up	Produced in each cycle in relation to action on alcohol and drugs, access to water, and reducing loss to follow-up for TB and HIV/AIDS treatment
Final interviews	22 final key informant interviews were conducted for the final evaluation using a semi-structured topic guide. Participants were chosen for their knowledge of the programme and included community partners (3), health system partners in the district and province (5), researchers (9), national collaborators (2), and international collaborators and advisers (3). Of the 22, 14 were female and 8 male.
Programme expenditure	Based on programme budget and further grants; assessed to inform on resource intensity of VAPAR
Stakeholder mapping	Conducted by research team at the end of each cycle, mapping key stakeholders against influence over and interest in the focal topic
VAPAR workshop reports	Each stage of each cycle included workshops with diverse participants; their feedback and team reflections
Other team outputs	Public engagement writing (e.g. The Conversation pieces); podcasts by team; short videos; training manual; research ethics training for province; local radio series in local languages
*External to VAPAR*	
Official documents from the health sector	For example, the District Health Plan, Annual Performance Plan, Integrated Development Plans, quarterly and annual reviews
National/sub-national policy and strategy documents	Relevant policies affecting community health, such as the National Health Insurance (NHI) plans, PHC Re-engineering strategy, and policies relating to relevant other sectors, such as water, alcohol and drugs
MRC/Wits Agincourt Unit data and research	Other ongoing studies and outputs of relevance (e.g. data on clinic functionality)

### Data analysis

Data collection and analysis were continuous, collaborative and inclusive, with reflections at the end of each cycle to aid learning and inform improvements. Results were discussed with actors from local to provincial level, such as community groups, PHC supervisors, facility managers, community health workers (CHWs), front-line health staff, health programme and research managers and agencies in other sectors such as water and sanitation, housing and the environment. Quantitative data such as VA were analysed for trends in health indicators for different population groups. The bulk of the data was analysed qualitatively, however, focusing on understanding relevant changes, drivers, perceived impact(s) and inter-relationships, how they interact with context (social, economic, political and institutional), unintended consequences (including negative), and implications for future efforts to build similar learning platforms. 

### Limitations

The participatory approach enabled elicitation of approaches, mechanisms and outcomes, and was coherent with our overall approach. Risk of an overly optimistic assessment was mitigated by continuity of data collection and multiple sources; findings on impact on collaborators, for example, were drawn from repeated observations and interactions over time. The team also practiced reflexive and self-critical thinking, drawing on our own learning regarding the (re)construction of safe spaces for constructive, respectful reflection and critique. Additionally, the evaluation lead occupied an insider/outsider position – sufficiently embedded to have a good grasp of the programme and context, while distant from day-to-day operations for a neutral perspective.

## Results

In this section, we present the programme evolution to provide background for the theory of change domains analysis. We then examine how the context evolved, and whether predicted opportunities and challenges remained salient. We next turn to whether expected inputs were delivered, and whether and how expected mechanisms of change were triggered (considering key stakeholders: community members, CHWs, health staff and staff from other sectors, and researchers). We examine evidence for hypothesised outputs and outcomes and review unintended negative or positive effects. Finally, we present resources to support the work, together with stakeholder recommendations for future directions.

### A. Programme evolution

VAPAR was designed to be adaptive, and the approach evolved in important ways. Instead of the planned three cycles, five were progressed in total. After cycle 1, lasting 18 months as planned, the second cycle was disrupted by COVID-19. In early 2020, the programme was co-redesigned to make practical contributions in the context of a new, public health emergency. The redesign focussed on the role and functions of CHWs; seen as first line responders by service users, providers, planners and managers, but as insufficiently supported and resourced ^16^. During cycles 3 to 5, VAPAR retained this focus, and engaged with CHWs and community members, as well as with health system stakeholders at community, clinic, sub-district and district levels, moving towards a ‘training of trainers’ approach to empower and support CHWs with PAR skills for community engagement. During the fourth cycle, a CHW platform was also established to create a peer support forum and for regular engagement with supervisors. Cycles 3–5 lasted 12 months or less.

Cycles 4 and 5 also saw VAPAR working outside the surveillance area for the first time, providing training and support to other parts of the sub-district, responding to requests from sub-district managers, and working with provincial colleagues in research governance and strategy. Focal themes changed over the cycles, from water and alcohol and drugs, as chosen by the community representatives in cycle 1 to loss to follow-up for TB and HIV/AIDS, as chosen by PHC health workers and officials, CHWs and community members, and which was a consistent focus in cycles 3–5. Engagement with the health system and public authorities also shifted. In the first cycle, stakeholders were multisectoral, reflecting the choice of topics. During stakeholder reflections at the end of cycle 1 in 2019, provincial and district DoH officials recommended a reciprocal arrangement for subsequent cycles: integrating VAPAR into routine PHC planning and review, and with frontline health workers involved in VAPAR processes ^15^. After this point, there was a stronger focus on linking with district and sub-district. The programme remained open to opportunities and changing needs, including with provincial health research governance and strategy (during cycle 4) and in establishment of the CHW platform (in November 2022) ( Table 2).

**Table 2.** Summary of VAPAR cycles (Source: authors).

Cycle	Summary of participants	Duration/ date	Focal theme	Location/coverage	Summary of process	References
1	48 community members	2017–19	Access to safe water; alcohol and other drugs	3 villages in Agincourt DHSS area	Multisectoral local action plans developed and implemented with water and sanitation, basic education, South African National Council on Alcoholism and Drug Dependence and social development	12
2	54 community members	2019–20	As above, with co-re-design at onset of COVID-19	3 villages in Agincourt DHSS area	Cycle interrupted and co-redesigned to support district in early pandemic phase, which led to focus on CHWs and HIV/TB	C2 reports
3	9 CHWs; 6 community mentors; 27 community members	February–April 2021	LFU for TB and HIV/AIDS	3 villages in Agincourt DHSS area	Rapid PAR training package for CHWs developed and rolled out in 16 workshops	14; 16
4	54 CHWs; OTLs; 3 CHW mentors (from C3); 53 community stakeholders	March–May 2022	LFU for TB and HIV/AIDS	6 local areas in Bushbuckridge sub-district	41 workshops, alternating theory and implementation, using revised training manual	C4 reports
5	53 CHWs; 10 CHW mentors; 52 community representatives	February to May 2023	LFU for TB and HIV/AIDS	Bushbuckridge sub-district – reaching 5 more local areas	Training of trainers, with CHWs from C4 as trainers. Programme of 8–10 weeks, with one workshop per area of 2–3 hours each week.	C5 reports

### B. Context

Contextual challenges which the programme sought to address included lack of constructive engagement between communities and health system, and organisational culture issues on data-driven decision-making within the health system. Resources and opportunities included a progressive policy environment and a growing network of institutions collecting data on community health. The continuing development of NHI opened up spaces to reinforce the role of the CHWs, for example ^17^. Meanwhile, there was a continued expansion of research infrastructure by SAPRIN, the South African Population Research Infrastructure Network. VAPAR made modest contributions to their growth by supporting data integration ^18^, and engaging with the Limpopo HDSS site in 2022 (with a pilot adapted from VAPAR’s methods focused on NCDs). Support for research governance and strategy in 2022 with the Provincial Health Research Committee ^iv^ was a further contribution to expanding research capabilities in the province. These demonstrate some of the dynamic linkages between context and programme intervention.

Some challenges – such as top down/hierarchical governance, limited operational autonomy, and low accountability to service providers and users – link to limited ‘decision-space’ by local actors to use evidence for local change. These are perceived as relatively constant over the period and remain a challenge. Our decision space analysis concluded that while capacity exists in the system, accessing it is difficult. While lines of authority are well-defined, personal networks are important and expressed through informal coping strategies. There is limited formal external accountability to communities, and internal accountability which is weak in places for individuals and focused more on meeting performance targets set at higher levels and less on enabling effective local leadership. At sub-district and facility levels, constrained capacity was the dominant theme ^19^. In relation to the system operating ‘in the dark’ without using local data ^20^, lack of communication and trust between communities and authorities, and lack of power and representation of communities, are all areas that VAPAR attempted to address to some extent, although they remain problematic.

*Are we really able to confront the underlying system constraints, for example pushing back on the culture of blaming patients?* (KI 6)
*…operational issues still have to follow politics* (KI 15)

Elsewhere, KIs reported that researcher incentives remain highly driven by the funding environment, which has become more competitive. This means that time and resources to engage with data uptake locally remains under-resourced, compared to publishing - even as funders highlight the importance of ‘real world’ impact.

### C. Inputs

Inputs were conceptualised as support for co-production of timely and relevant local evidence on local health concerns and enabling exchange with stakeholders within the health and public administration system. In this section, we look at these and reflect on the programme adaptations in a changing context.

***i. Relevance of data***

In each cycle, VA data were assembled with community-generated PAR evidence on the focal themes. These ‘inputs’ were presented in evidence briefs ^v^. Health systems officials appreciated the combination of statistical data on local burden of disease with evidence on the human experience of the burden, for example through Photovoice data. Nevertheless, the focus on social determinants in cycle 1 presented challenges to DoH stakeholders, as the topics, while recognised as important to health, lay outside their sphere of influence.

*In the early stages, work pointed to the high levels of children deaths in the community. There seemed to be progress in hospitals, and it was a shock to find so many children dying in the communities. The original excitement for us was being able to probe the reasons why this was happening. Agincourt offered a real treasure trove of data. But in the process, the programme responded to community demands and moved away from these under-five deaths. It focused on alcohol and drugs and water. These do impact on child deaths relating to malnutrition, diarrhoea and maybe pneumonia. But there was a disappointment that it moved away from our original excitement about understanding child deaths better* (KI 2)

In response, the focus on CHWs was seen as a clearer fit, reflecting the views of service users and providers ^21^ and linking to health sector priorities, such as Ward-Based Primary Health Care Outreach Teams (WBPHCOTs), the Medium-Term Strategic Framework and NDP Implementation Plan 2019–24, onto which the Mpumalanga DoH strategic plan 2020–24 is anchored. The provincial annual performance plan (APP) and district health plan (DHP) all highlight quality of care and CHW competencies in community mobilisation ^vi^; and all stakeholders recognised the importance of community voice in service planning and delivery for PHC, viewing VAPAR as supportive in this process. The focus on CHWs as the first line response of the system and a vital connector of communities to system was also universally acknowledged, with recognition of need for support.

*CHWs are key to government policy now. There is a focus on standardised training and connecting community with services, so the current VAPAR model is spot on* (KI 15)

***ii. Relevance and adaptiveness of model***

VAPAR was perceived by health system and wider public administration stakeholders as an innovative model, bringing together groups who generally work in siloed ways.

*It was unique in that it took participatory research into an institutional environment, the health system – not just focussing on communities. That creates a new dynamic. PAR in communities does not necessarily generate sustainable change* (KI 6)
*I was impressed to see how many people were present in that meeting – so many stakeholders under one roof. Community members, traditional authorities, and other departments, which was not happening before. It really tried to bring together different stakeholders, which was impressive…Other researchers come, collect data and go. But this one was very different* (KI 3)

The VAPAR model was designed to evolve through the cycles in response to lessons learned, dialogue with partners and steering committee members, and context change (such as COVID-19). Iterations were characterised by a growing awareness of the need for the approach to be relevant and pragmatic if it were to be sustained and institutionalised. The shift of focus of engagement from provincial to district, sub-district and facility reflected this awareness and an analysis of where change was possible ^19^.

*The water presentation when the community members presented was powerful. But we were too ambitious – raising community voice, doing intersectoral action, acting at all levels of the health system. It was too much* (KI 7)
*The shift to the district level has been successful – more of an open ear, fewer other distractions. VAPAR was able to feed into monthly meetings etc – a good move as it made research more tangible to district managers, who are responsible for service delivery and service providers* (KI 2)

Capacity building was added to the theory of change in cycle 3, reflecting CHW engagement and recognition of the need to build PAR skills in the system. This fed through into participants becoming mentors and trainers. The research team also reflected on their roles, and the tensions between an embedded and sustained process and one in which external researchers are involved. While sustaining the process seemed to be culmination of the shifting ownership, the team were seen as ‘neutral arbiters’ creating spaces and processes for data-driven dialogue, action and learning; articulated in terms of the ‘triangle that moves mountains’ ^22^. These adaptations are judged to have been successful and appropriate; however, some KI reported difficulties grasping the programme’s focus (VAPAR being not only complex but also changeable).

***iii. Quality of engagement***

Engagement grew in the sub-district and district over time. Following the reiteration of cycle 1 to 2 in 2019, the provincial DoH endorsed embedding the intervention into routine PHC planning and review at district and sub-district levels, including District Health Management Team (DHMT) meetings ^15^. In 2021, the district requested the CHW training be rolled-out across the district. Regular attendance at workshops by the Outreach Team Leaders (OTLs) and clinic Operational Managers (OPMs) also demonstrated support and commitment from facilities. CHWs furthermore appreciated support received from leaders. There was a trade-off, however, in that some of the wider multi-sectoral relationships built during cycle 1 were less engaged later in the programme. Some KIs also highlighted the time that it took to build these relationships and engagement:

*Outreach Team Leaders (OTLs), CHW coordinators, Operations Managers (OPMs), Traditional Healers, Clinic Committee members from the sub-district levels and community members were engaged…VAPAR’s strength is in connecting different stakeholders and making them understand and appreciate each other’s work* (KI 21)
S *ometimes it was hard to explain our role, that we don’t bring in financial resources but something more intangible, especially for the district health system stakeholders…Maybe it takes a long time* (KI 1)

Some 240 community participants engaged across five cycles. In the first, community members were approached from the pilot. They included people affected by the nominated topic, but also community leaders, with appropriate management of group dynamics and use of local languages and local meeting spaces to support inclusion and participation, and with control progressively shifting to participants. Community partners also expanded the participant base, directing recruitment as relevant for the themes selected – for example, youth were engaged to address alcohol and drugs, and women to address water access.

In cycle 1, community representatives were active in local meetings as principles of inclusion, voice, power-sharing were regularly discussed. As the process expanded into the health system, dialogue with officials, health workers, planners and managers was emphasised. The VAPAR core team, based at the South African MRC/Wits Agincourt Public Health and Health Transitions Research Unit, was seen as strong, however, the model was also perceived as complex, and subscribing to non-positivist analytical paradigm, which created some barriers to engagement by other researchers at the unit.

*Communication was key. In VAPAR, everyone is encouraged to participate and contribute to decision-making. This made CHWs understand their value and that together with other stakeholders, they could make a difference* (KI 21)
*It was a very strong team. It was very clear about theoretical and conceptual issues that are not always well understood. But the approach was hard to keep in your head* (KI 8)

### D. Mechanisms

In relation to mechanisms of change, three channels were hypothesised: greater confidence in and commitment to co-producing and using evidence by all stakeholders; improved relationships and trust between communities, researchers and authorities; and increased motivation and capacity for community involvement. Ex post, another mechanism was added (as emerged during cycles 3-5 with CHWs) related to increased role clarity.

***i. Confidence and commitment to producing and using evidence***

Among clinic, sub-district, district and provincial staff, there was increased recognition of the value of community evidence for decision-making processes. During the programme, more systems stakeholders joined in co-authorship of published papers (around one-third of the papers were co-authored by health system partners to date, with these more common later in the programme). However, constraints remained in terms of systems stakeholders having time to engage, as well as being narrowly focused on core performance targets. The change of theme also hampered longer-term engagement with stakeholders from other sectors and municipalities.

*We helped to link the CHWs with the clinics and with communities. There was an increased awareness by staff of community needs... The DoH were not previously using research data, but started to realise its value* (KI 1)

In relation to communities and CHWs, there was a clear growth in skills to assess problems, identify root causes, analyse stakeholders and plan local action, following the tools provided and adapted by VAPAR ^vii^. CHW’s reported a ‘triple’ benefit of peer support, acceptance by communities, and recognition by the system. CHWs also reported how their agency was reworked in more empowered ways, through the regular learning spaces bringing together peer and non-peer perspectives. CHWs reported the use of specific tools elsewhere in clinic operations, especially the problem tree tool, for example:

*You can’t solve a problem without knowing where the problem comes from. So, the method is relevant. People love this method – identify the main problem, who is involved, finding an approach to deal with problem. Previously simple assumptions about causes and answers are challenged. The problem tree is most useful – who is contributing to an issue?* (KI 17)
*I have gained a lot of confidence. Previously I was afraid to speak, even to the OTLs [outreach team leaders]. Now I know how to approach a problem, using the problem tree* (CHW, final workshop)
*CHWs actively participated…making suggestions and proposing actions and activities, as well as voicing their views and opinions. They also displayed confidence when doing the Venn diagram as they are familiar with this participatory tool and could guide other stakeholders and lead this activity during group sessions* (Cycle 4 report)

In relation to the research community, including at Agincourt, there has been some uptake in interest in PAR methods and establishing platforms for data-driven learning. VAPAR was based in the public engagement office of the HDSS, and regularly shared its methods and findings with other researchers, although methodologically, it was an outlier, confronting some degree of epistemic silos (most research at the site being more positivist in approach although a health systems theme has endured for many years).

*It is a challenge convincing PIs – an epistemological challenge. HPSR is the Cinderella of public health, which is itself a Cinderella in South Africa* (KI 4)

***ii. Improved relationships and trust***

Building relationships and trust was at the heart of VAPAR’s approach, and considerable gains were reported. These included between communities and public authorities, between CHWs and health staff, between levels in the health system, also between wider stakeholders during multisectoral work, and between the research community and health system actors. In relation to community stakeholders, VAPAR provided stable ‘safe spaces’ for evidence, dialogue, learning and action which allowed a transition from initial tension and blame to willingness to seek cooperative solutions ^23^.

*If you give the community opportunity to raise its voice, because of the challenges in rural communities in infrastructure etc, you often get an angry voice. And angry voices don’t always create positive change. Now it seems that community feel better able to express themselves in a more collaborative way – people can sit around the table and hear each other* (KI 2)
*There have been a lot of service delivery protests in communities, but they did not accomplish much; everyone realized that it is time to shift our ways of thinking and initiate dialogue, unite, and collaborate and create sustainable partnerships to solve community problems* (Community stakeholder, reported in 15)

VAPAR also helped bring together stakeholders from multiple levels of the health system and across sectors (especially in cycle 1), which is rare in routine governance systems. Participants from health and other sectors appreciated this and gained some relationships.

*We struggled to bring different role players into one room, and VAPAR has greatly helped with that. Relationships with other governmental entities have been developed, a great benefit for us* (KI 2)
*This programme was important for me. I got insight on how other government sectors operate and working together with communities and other sectors was empowering. I also learnt how to engage service users in decision making and planning* (Government stakeholder, reported in 24)

For the CHWs, most of whom were previously home-based carers and transitioned into CHW roles often without formal training or guidance, the VAPAR training is reported to have helped clarify their role – between peers, among clinic staff, and in communities. Engaging in the process helped CHWs build trust and core PHC competencies, as well as break down professional hierarchies.

*VAPAR has taught us team building, has taught us love. We are able to adapt to every community now* (CHW, final workshop)
*Some new dynamics were observed with regards to the relationship and interaction between different cadres, which may be interpreted as improved relationships, tolerance and acknowledgement between the CHWs, and other health profession ranks within the health system. During previous workshops, CHWs would sit on the opposite side of the venue than the OPMs and OTLs. During this workshop, the different job categories were not seated apart or on different sides of the room but were integrated… The workshop was also characterised by free and open communication by all stakeholders’* (Cycle 4 report)

This process reflects the facilitation skills developed by the team.

*We created safe spaces, showing ourselves to be trustworthy. We listened and showed that every opinion deserves respect…. We paired the CHWs so they could learn from one another and put them in charge of the process. They had knowledge but lacked confidence and were taken for granted. They can do a lot, but they need support. And they want to learn!* (participant in final workshop)

KIs noted, however, that trust has to be continually built and that *‘increased trust by a municipal manager coming to the meeting and being honest but doesn’t equate to generalised increase in trust’* (KI 7). Finally, some deepening of mutual knowledge and relationships was noted between the research community and health system actors, increasing the linkages between the HDSS and different levels of the health system.

*Even in Agincourt they are still benefiting from relationships that we have built in the province and district. They can benefit from these long term* (KI 1)

***iii. Increased motivation and capacity for community engagement***

Among health system actors and public authorities, engagement with VAPAR was seen to reinforce the value of community engagement.

*It did add an awareness within the people that we interacted with in the DoH and other agencies that community voice mattered, and to that extent it was good and unusual* (KI 7)

Most significant was the impact on CHWs, whose capacity to work more systematically with community members to problem solve was highlighted in evaluations of cycles 3 and 4 ^14^. They report improved skills in public speaking, problem solving, collaboration and facilitation. This was supported by observations from health managers, who reported that CHWs were spontaneously using the Problem Tree tool in practice to identify and address technical issues. In addition to capacity, CHWs were given a crucial sense of recognition and hence self-confidence. This derived not only from the initial training, but also from taking roles as mentors to the next cohorts in cycles 4 and 5, as well as adopting roles as trainers in cycle 5.

*We have observed a noticeable enhancement in the ability of CHWs to facilitate meetings and to communicate with stakeholders after the workshops that were done during the last VAPAR cycle* (KI 2)
*Through the training, they got to know that they are the ones who know their patients very well. They can help identify the right approaches to solve problems... They (CHWs) have the power to change things. They have gained in confidence. VAPAR was interested in them* (KI 16)

At community level, an increase in skills and confidence to work together and with other representatives to address their challenges was reported.

*I have learned how to engage with people, I now realise I can speak freely, connect with people I didn’t know, even people in higher power* (KI 16)
*From cycle 1, the community learned how to problem solve, from the individual to community level. They considered what they themselves can do, not just blaming others… They are more likely to talk to providers, authorities, rather than protesting. They are more confident to approach providers* (KI 17)
*The process was reported as valuable and significant as it created an opportunity for communities adversely affected by lack of water to connect and constructively and actively engage with authorities/ service providers in appropriate and accessible venues… Dialogue between communities and government stakeholders helped to bring positive change, as community members began to recognize their own expertise, and this contributed to improved interest, commitment and buy in of multiple stakeholders… Local community members reported improved awareness and understanding of the various stakeholders within the water supply chain, and the bureaucratic procedures among government departments’* (KI 17 ^24^).

Researcher capacity was not in the initial theory of change, however emerged as an unintended positive impact. Several of the team developed significant skills in facilitation, community engagement and training, as well as post-graduate qualifications ^viii^. This will have ongoing benefits for the HDSS, although it will be important to keep investing in the community engagement team to retain that capacity. Some lessons learned in relation to mechanisms are summarised in Box 1.

Box 1. **Transferable learning on mechanisms (This box has been reproduced with permission from Source: ^25^ under a Creative Commons Attribution 4.0 International License, which permits use, sharing, adaptation, distribution and reproduction in any medium or format, http://creativecommons.org/licenses/by/4.0/)**

*•*   Regular community engagement builds strategic, analytical and public-speaking skills and confidence;

*•*   Regular engagement between communities and authorities, fosters awareness, mutual understanding and trust;

*•*   ‘Safe spaces' outside institutional processes to connect with and understand other agencies and communities are valuable;

*•*   Spaces close to implementation contexts support inclusivity, managed expectations, reinforced principles, and a process owned and controlled by those involved;

*•*   Processes framed as shared endeavours can deepen engagement, ownership and understanding;

*•*   Processes need time to build and maintain constructive, cooperative relationships and trust between sectors;

*•*   Monitoring is effectively done by those closest to the issue;

*•*   Recognition of roles of mediators is important – facilitators between communities and authorities with two-way communication “bridging cultural and power gaps”;

*•*   Incentives and renumeration require careful consideration as positive reinforcement to sustain the practice

### E. Outputs

Expected outputs included establishment of a learning platform/space, ownership and uptake of locally relevant evidence, and collective action and learning.

***i. Establishment of learning platform***

Engagement with the platform was strong, especially from local health system stakeholders, CHWs and community members. This led to the two-way integration of the process into the DHMT, and health workers and officials joining the VAPAR cycles ^15^ reflecting established relationships and perceived value. Stakeholders appreciated being connected to new constituencies, such as traditional leaders, parastatals, non-governmental organisations (NGOs) and community members ^15^, although some gaps were noted (e.g. less involvement of political leaders). There was appreciation of the process and shared leadership within it. VAPAR also opened new spaces and platforms for participants. For example, building on their skills, CHWs were asked to speak in other fora on health messages (for example, in churches and to traditional leaders).

*It gave me a platform to stand in front of big managers… I was also given space by churches, where some pastors were giving wrong messages… There was also recognition by the induna [traditional leader]* (CHW, final workshop)
*VAPAR also related to traditional leadership well. Others go straight into communities, but VAPAR has respected traditional authorities and engaged with them. That is how you get good information* (KI 3)

The CHW platform may also bear fruit, although it is too early to assess. It recognises that most CHW participants were neither aware of nor supported by outreach policy and strategy. A sub-district based CHW platform was seen to facilitate communication between health managers and CHWs and support recognition of CHWs. The group included one CHW representative per local area, one unfunded CHW (to represent those still not absorbed into the payroll, an ongoing challenge), the CHW coordinator, and a VAPAR representative. The aim is to meet quarterly for debriefing, problem solving and peer support. In the first meeting, poor data capture was discussed, with action agreed to strengthen practices in clinics. The relevance of the CHW platform was highlighted by KI:

*They (CHWs) have a lot of challenges and don’t have anyone to talk to who understands their issues. There is no regular forum …The supervisor attends and can advise, she can also bring stakeholders to the meeting, if relevant* (KI 15)

***ii. Generation and uptake of local evidence***

The range of evidence co-created by VAPAR was impressive, with products aimed at academic and non-academic audiences (including papers, podcasts, webinars, radio series and press articles ^ix^). Community topics and data on alcohol, drugs and water were relevant and connected to social determinants. Analysis of the MRC/Wits Agincourt VA data found that over 75% of deaths were attributable to at least one community-nominated risk factor ^26^. The programme also invested in the wider research ecosystem in the province, supporting the Provincial Health Research Committee to develop a governance strategy and providing research ethics training ^x^, which supported the committee to gain Research Ethics Committee status. However, the research committee still faces constraints to implementation, including a lack of resources and limited staffing, according to KI.

*The province wasn’t able to follow up on information from research. Once they [researchers] get their results, they are gone. The next thing you hear is publication. We wanted to find a way to hold researchers accountable. So, we developed a research uptake model – aiming to work with projects to engage wider stakeholders… Now we have that model and are trying to find a way to take it forward. The idea came out of interaction with VAPAR; we have had continuous engagement through the project... We also realised that we needed to develop local research priorities, so that research can be more useful. The leaders from VAPAR helped us to think this through* (KI 3)

***iii. Collective action learning***

Each cycle (other than cycle 5, which was undertaken as ‘training of trainers’) generated agreed local action plans, which were implemented and monitored. An appreciative approach to monitoring avoided unnecessary and unproductive punitive focus, to identify system constraints where actions were not completed ^25^. Published papers provide detailed accounts of these. Here we make some cross-cutting observations, partly important for outputs and outcomes, as well as for binding the groups through demonstration of agency and self-efficacy – learning by doing.

There were different forms and extents of action, and action plans evolved over the cycles. In the first cycle, actions requiring resources and/or higher-level engagement were less likely to be completed. For example, for the agreed water actions, three out of seven items were achieved ^24^. DoH and education-led actions were not completed as they lacked the ability to mobilize resources, including time, to execute the actions, despite initial willingness ^24^. For alcohol and drugs, of the four partially completed action items, two involved actors at national level (drug laws and compliance in taverns), one depended on departmental funding (social support services dissemination), and one was longer-term in nature, and subject to shifting priorities and spending (community rehabilitation centre) ^25^. The action-item achieved was the mapping of substance abuse hotspots. This was led by youth representative supported by researchers, with an arguably higher ‘stake’ in the process, coupled with a short-term, relatively achievable outcome ^25^.

*… one action-item was not achieved: health sector support for schools. This activity had been displaced owing to multiple, competing priorities among nurses at PHC level... it amounted to encouraging nurses to adopt schools as part of their social responsibility, which was not feasible in practice. Coordinating with governmental priorities was identified in response, as critical to support action and impact* ^25^.

Across cycles, as ownership transitioned, action became markedly localised; closer to communities, and within the control of those committing to them. Action also became more closely aligned to policy priorities, to incentivise and contextualise follow up. For cycles 3 and 4, partial to total achievement of the action plans was reported ^16^.

Activities planned and conducted by the CHWs in cycle 3 included setting up or revitalising patient support groups, carrying out health talks in clinics and during home visits on different diseases as well as disclosure, presenting at traditional healers’ fora, and conducting door-to-door awareness campaigns on TB. Cycle 4 actions reflected those in cycle 3, and also included taking on direct observation of treatment for some patients, educating family and patients on disclosure. OTLs, OPMs and community stakeholders confirmed that all actions had been completed (or were ongoing, by nature). Additional challenges, for example on social support, were highlighted and options for addressing them discussed. CHWs also cascaded training on PAR to colleagues.

*They would come up with unrealistic plans at the start but as we proceeded, they started to develop more effective and collective plans… more realistic and feasible* (KI 1)
*We not only developed action plans but also trained colleagues in the methods, and gave talks in churches. We also came back together to reflect on our success and failure. For example, why weren’t men coming to our support groups? How could we bring them in? We shared solutions across our groups* (CHW, final workshop)

CHWs also reported working with other groups, such as the Department of Social Development, also with clinic committees, schools and churches. They showed creativity in solutions, for example, finding ways of packaging pills for those who have not disclosed, and promoting home gardening to help with nutrition (final workshop). At the same time, the multiple constraints to CHW action and support were also highlighted, including lack of transport for OTLs, areas uncovered by CHWs or where CHWs are not on payroll, CHWs being employed on short (six-month) contracts, lack of standardised training and tools, lack of clarity on job descriptions, lack of airtime to be in touch with households and supervisors, long distances to reach villages and lack of transport for CHWs, low remuneration, high workloads and belittling treatment in clinics ^14^. Each cycle had reflexive elements to give space to reflecting on the actions and learnings from them. Some KIs noted that more strategic reflection requires longer term engagement.

*I am more cautious about the action loop – the programme identified priorities and actions but were we learning from the implementation of actions with communities? Maybe that needed more iterative cycles* (KI 6)

### F. Outcomes

Posited outcomes were grouped into five areas and as shorter to longer term: improved health service processes and organisation, policy implementation, resourcing and delivery; improved health behaviours and outcomes; improved understanding of and commitment to equitable health priorities by local system actors; sustained and legitimate learning platform; and transferable process and shared learning.

***i. Service organization, resourcing and delivery***

Improvements in service organisation and delivery were noted and linked to VAPAR. These included improvements in delivery of water to communities in the study site, which was a perceived outcome by community-based interviewees ^15^. Improvements in law enforcement with regards to trading hours of taverns, as well as noise levels and environmental pollution, were also observed by community-based interviewees. KIs narrated how mindsets were changed as local councillors and senior police officials became aware of community concerns through the process ^15^.

*Concepts and perceptions have changed. People were into drugs, especially youth but now it is quiet, you don’t hear noise. There is limited use of drugs. The action plans developed with the community helped to influence the community members. The taverns are now regulated, and they do not open throughout the day. Before VAPAR, people were throwing and dumping waste everywhere but now we have black bins which they come and collect. Community awareness was raised on the dangers of pollution (e.g. dumping waste everywhere, like children's diapers) and drinking contaminated water. VAPAR changed the mindset of the community* (KI 20, community stakeholder)

KIs reported that higher COVID-19 vaccination rates in the sub-district were attributable to newly empowered CHWs, although other factors were likely to be at play.

*With COVID-19 vaccination, BBR sub-district was doing well, even NDoH wanted to know how they managed this. BBR showed good practice, the CHWs were pro-active and well trained. The way they (CHWs) articulate themselves. We asked how they reached people – based on this, they shared the VAPAR program information. All indicators, childhood and causes of death – it was taken to other sub-districts to align their plans accordingly* (KI 10)

The cycle 3 evaluation identified process changes expected in the medium term ^16^. They included: early identification of challenges; problem solving/resolving challenges; shifts in health system response to community needs; community attendance and collaboration on health awareness days; increased uptake of health services; gaps in health system identified and addressed; increased efficiency in patient care; and improved upward referral within the health system, with potential for improved health outcomes. Some areas were identified as having improved, including data quality on HIV/TB loss to follow up and streamlining tracing processes, due to the actions planned and undertaken by CHWs. For example, in one clinic, there was concern about high loss to follow up. On investigation using the problem tree approach by the CHW and clinic operational manager, it was discovered that the problem was poor recording, and changes were made to routine clerking that avoided unnecessary follow ups, and the tracing process was redesigned. The system gained efficiency, avoiding time wasted chasing patients not in fact lost to follow up, and a general increase in recognition and functionality of CHWs.

*As CHWs, we now use skills we got from VAPAR daily when we go to visit households. We are now confident enough to do our jobs and it is much simpler now because even now the staff at the clinic understand and support us… I have learned communication and engagement, with different stakeholders and I am now able to identify and engage with different stakeholders in my community* (KI 22, CHW)

***ii. Health behaviors and health outcomes***

Some changes are evident in mortality and morbidity in the programme site 2017–22, which likely link to COVID-19 – for example, VA data show an increase in deaths from acute respiratory infection (from 5.7% in 2019 to 19.4% in 2021), which may be linked to reductions in hospital care-seeking, and increased use of traditional practitioners, as suggested by circumstances of mortality categories (COMCAT) data. Measurable impacts on health outcomes were not realistic in the short term, however, and considering the range of health issues and social determinants addressed. Over time though, community awareness, education and engagement were identified as important means to improve outcomes through improved behaviours and services ^15^. CHWs also report saving lives locally after engagement in the programme, including through role clarity, increased recognition, and more effective communication with clients, and improved services, which should yield further benefits over time.

*My behaviour also changed because of VAPAR, because I no longer feel undermined. The community shows their interest and willingness to be assisted by CHW* (KI 21, CHW)
*I called and cried. I said I am crying for your life I want you to come here. And then he came [for TB testing] …We are able to do an impact in our communities. Should the nurses conclude that this person didn’t have TB then maybe that person would have died but… I saved a life* (CHW, final workshop)

***iii. Equitable health priorities***

Stakeholder mapping suggested that groups with which VAPAR had engaged most closely in each cycle had increased in interest and investment in the topic ^19^. In some cases, like the CHWs, they were also seen as having increased in influence, though this dimension is stickier than interest (and in the CHW case they were starting from a low base). KIs report impacts from the pilot and in cycle 1 on under five mortality – that the visual evidence of living conditions sparked shock and changed the way that nurses did health education. KIs also reported how the process helped change community mindsets *,* engaging actors such as local councillors, and disseminating clear messages to address local concerns over alcohol, drugs and environmental health.

*Showing photos that the community had taken of children at risk in the community and showing them to province and district people and them not believing they were from South Africa – that led to action. That led to changes in how nurses in clinics did their work on health education* (KI 7)
*Information was disseminated to the government and the ward councillor, because of VAPAR, the ward councillor is trying to address the challenges and concerns in the community. There is a dialogue with municipality officials…There are improvements in the community, water infrastructure has improved* (KI 20, community stakeholder)

In cycles 3–5, role clarification and engagement with OTLs was an important benefit reported ^14^. Within communities, CHWs have been helped to build skills in community mobilisation as related to health education, treatment support, and referral. The rapid PAR training was aligned to core CHW competencies for PHC and was certified in collaboration with the provincial DoH. While there are clear benefits to communities and clinics, some tensions remain between community- and clinic-based work of CHWs.

*VAPAR has changed the way the clinic staff, and us (CHWs) and the community view us. We were undermined, but now CHWs knows their scope of practice. Things are much clearer for us, which is good. VAPAR helped CHWs to see their importance and the positive contribution that they render to the community. CHWs has been empowered, they are now able to identify different stakeholders without any fear, and they do feel limited* (KI 22, CHW)
*The communities also benefited too – they get attention from highly skilled CHWs, thanks to VAPAR. CHWs even help with them getting medicines. They can ask CHWs questions too, e.g. if meds look different, you can ask the CHWs for clarity. The clinics also benefit – e.g. when they are short of staff, CHWs can help getting health files out in the morning, doing BP, doing health talks* (KI 17)
*The local system managers were more responsive, the OTLs too. They weren’t getting good reporting from CHWs before, this helped them get better reports from CHWs, and more clarity on own role. Lots of theories about WBPHCOT in policies, but they are not necessarily trained or supported* (KI 17)

***iv. Sustainability***

There is evidence of increasing integration of VAPAR into routine systems in the district since 2021, including with DHMT meetings, engagement of sub-district staff in VAPAR activities and growing participation of CHWs and clinic staff in training and other activities. However, contextual factors have sometimes been unfavourable: the DHMT meetings became irregular during COVID-19, limiting VAPAR’s engagement in them. Following the CHW training, health managers recommended that CHWs should present in PHC meetings, where performance indicators are discussed, which would enhance the sustainability of the learning activities, if adopted. Other activities were also collectively identified (cycle 4 reports) to continue momentum, such as rolling out further training in each area, with OTL support and using the VAPAR training manual; using trained CHWs as mentors in community mobilisation for their peers; and ensuring that CHW reports are incorporated in monthly health facility reports (to elevate their feed-back to senior management). Fundamentally, sustainability lies in the skills acquired by participants to date, according to some KIs.

*It would help to keep us more grounded. We often talk about the reasons for poor performance, but this is based on assumptions about what is happening at the grassroots* (final workshop participant)
*In the local area, it can be sustained with the leadership of OTLs, working with CHWs who are now trained. They can sustain the process, whatever the topic* (KI 17).

Most notably, following Cycle 5 in 2023, there was demand from the province and districts to scale-up the PAR training across all three districts in the province: Ehlanzeni, Gert Sibande and Nkangala. The team secured additional funding to respond to this request and are progressing cadre- and condition-specific adaptions of the VAPAR CHW training across the province. This additional dissemination and engagement seeks to embed practical solutions for community participation across practice settings and share learning to facilitate policy and strategy commitments to participation in health.

*The VAPAR platform and process has been well-received by all stakeholders and a strong partnership has been forged, with notable positive outcomes. Of note, the training of CHWs in Bushbuckridge sub-district…has resulted in significant and notable improvement in the capacity and performance of this cadre of health workers…the Ehlanzeni health district management team has noted the positive outcomes of the VAPAR programme... and request the learning and processes from this programme to extended* (scale-up request, district DoH)
*The VAPAR programme has been implemented in Bushbuckridge sub-district, Ehlanzeni district. Reports and feedback from this area demonstrate significant capacity building and the establishment of conducive relationships between local communities, community structures, and health system stakeholders…As such, we herewith request the VAPAR researchers extend the learning from this programme to other districts in the province, to further share the process of community engagement and other outcomes with health programme and system managers* (scale-up request, provincial DoH)

As an institutional base, the Agincourt HDSS provides a potential long-term home for the research and data component of the VAPAR ‘triangle’ (of community, health system and researchers) and is developing other programmes that could benefit from and take forward the VAPAR work. However, KIs highlight that the activities remain dependent on researcher facilitation and the modest resources needed to support activities. Some KIs also highlight the importance of champions. While some have emerged at different levels (province, district, sub-district and the research environment), it is not clear whether these individuals have sufficient influence, especially in a time of resource constraints, as currently faced. Certainly, there was insecurity among some KIs about what would happen in the absence of the current programme support and a sense of dependence.

*Real institutionalisation takes time and is not driven by outsiders but by those in the system doing things in a different way. The system doesn’t change quickly unless there are high-level champions* (KI 6)
*We don’t want to have you and then you leave with everything (knowledge, attitude, skills) and we are back to zero* (KI 10)
*… it will require financial support to continue* (KI 12)

Others highlighted that the programme had organically found its way to a good model that fits the policy context and current needs, which aids chances of being sustained, although the context remains inherently uncertain. KIs also pointed to the challenging context for sustainability.

*It is not clear if that will continue beyond a structured research process. It may do, as doors have been opened, but that is certainly not guaranteed. I have seen that in other projects – transfer of skills is not sufficient to get people on the ground to continue. It may not have priority over APP targets etc* (KI 2)
*We all need to learn about how to institutionalise changes from PAR; it is very challenging, even if there is lots of energy at the grassroots level in projects* (KI 6)

***v. Transferable process and shared learning***

The programme has been active in sharing its approach and lessons at several levels, including with other researchers at the MRC/Wits Agincourt HDSS and SAPRIN, within the district and province, more broadly in South Africa and the region, and internationally, although the impact of these activities is not easy to track and is inherently unpredictable. Within the research centre, VAPAR shared its approach with colleagues through talks, as well as supporting staff development, and supporting Agincourt to become a WHO collaborating centre for VA. There is evidence from some recent research projects of a growing focus on connecting data with communities and services (for example, MADIVA ^xi^). Within the district and province, the VAPAR programme expanded to cover the whole sub-district in cycle 5, using a ‘training of trainers’ approach focused on PAR. As noted, above, in 2023, roll-out to the whole province was requested by senior management. The CHW training manual has been refined with each cycle to support this. Within South Africa and the region, links have been made with other health policy and system research (HPSR) centres, where a community of practice is under discussion.

Furthermore, a pilot was initiated in 2021 in the neighbouring HDSS in Limpopo province, to test transferability of the VAPAR model, focussing on non-communicable diseases (NCDs), and feeding the action plans to the district. A further pilot was undertaken in Rwanda in 2021–22, working with the University of Global Health Equity and focussed on CHWs engaging with NCDs for the first time. Others have used the CHW training manual and adapted the tools for use in their own PAR (for example, ReBUILD in Sierra Leone ^xii^). VAPAR’s methods were also adapted for three recent NIHR research projects ^xiii^. Finding appropriate institutional bases to scale-up such work is one topic raised by KI. The SAPRIN network which links the HDSS sites in South Africa is another possible platform, although this would require nodal director buy-in and prioritisation, according to KI. VAPAR contributed to the development of the SAPRIN network through data integration support ^xiv^.

*SAPRIN requires all nodes to have community engagement processes and CABs (Community Advisory Boards). VAPAR may be something that we can introduce in that context, in a standardised way of exploring taking research findings a step further, to generate action (elevating findings). That additional step happens rarely* (KI 14)

Unusually, learning also travelled south to north, with the methods being adapted to support research and system strengthening in Scotland, to address issues such as food poverty ^27^, and action on smoking ^xv^ ^28^. The PI has also taken lessons to the Scottish Parliament Cross Party Group on Improving Scotland’s Health. In 2020, a secondment into the UK health system (NHS Grampian) during COVID-19 allowed VAPAR experience to be fed into adaptive systems learning. Exchanges between Mpumalanga DoH and NHS Grampian were also organised in 2022, with community empowerment and learning health systems included in NHS Grampian’s 2022-28 strategy ^xvi^. In terms of training, VAPAR has transferred learning through activities and materials, deployed nationally and internationally. Materials include the CHW training manual in English and Xitsonga ^xvii^, a set of introductory videos for PAR practitioners ^xviii^ and training modules on research ethics ^xix^. The programme has also contributed to the HPSR module, now mainstreamed in postgraduate global and public health training at the University of Aberdeen for nine years ^xx^. Training in participatory methods was conducted for Rwandan and South African researchers in 2023, and VAPAR has supported 13 Masters dissertation placements and one Doctoral student ^xxi^.

There have been academic outcomes. 28 academic papers have been published to date ^xxii^. These include methodological reflections on VA and PAR, also thematic work on water, alcohol and drugs, child health, HIV/AIDS, and NCDs. They also share findings of cycle evaluations as well as reflections on health system issues. They reflect a wide authorship, which initially was academically focused but from 2021 onwards has included greater representation from health system partners. The team has been active giving conference talks and presentations in South Africa, the UK and globally (including the Global Health Systems Symposia 2018, 2020 and 2022) ^xxiii^. VAPAR also gave evidence to the Ministerial Committee on Mortality and Morbidity in Children Under-5 in South Africa in 2019. The team communicated with diverse audiences, including public engagement through articles for The Conversation ^xxiv^, case studies shared through Participedia ^xxv^, podcasts ^xxvi^ and radio series ^xxvii^; articles for CHW Central ^xxviii^, webinars, website and social media.

An important methodological contribution was the extension of classifications of deaths in VA to account for social and health systems circumstances, which has been integrated into standard interpretation and mortality classification systems and international standards. During VAPAR, these have been further refined drawing on VA data from across South Africa, in collaboration with SAPRIN ^20, 29, 30^. The research team has incorporated COMCAT into open-source software to analyse VA data that is freely available and COMCAT has been taken up in a national cause of death study in South Africa, and in India, Malawi, Mozambibque, Nepal, Pakistan, and Saudi Arabia ^23, 31^. Team members also contributed to VA updates in 2012, 2016, 2022 and 2022, led by WHO ^xxix^, and to Wits School of Public Health being established as a WHO collaborating centre in this area, as well as to an international VA working group, established in 2021. The programme has also contributed to the field of PAR, through lessons learned in relation to its implementation (see for example the transferable learning lessons highlighted in 32, the practice framework developed ^33^, and contributions to wider thinking on PAR and empowerment ^33, 34^ and learning health systems ^35^.

Conceptual work was also progressed to support institutionalising participation within decentralised health systems. Bossert’s decision space framework, applied to Mpumalanga DoH, identified decentralised spaces and areas for growth. This informed the process; whereby learning spaces were developed to find and amplify less-heard local voices ^36^. Fox’s strategic social accountability concept further informed VAPAR processes encouraging ‘state-society synergies’; whereby partnerships were advanced across communities, the health system, and between peers and non-peers, building local actor power overall ^25^. Following the final cycle, the team analysed the process using Popay’s community power-building framework. Here, collective capacities for joint action were seen to *emerge through collective adaptive processes*, creating spaces where people produce and use evidence to make decisions, and a practice framework was proposed to: (1) build community capabilities, (2) navigate social-institutional contexts, and (3) sustain authentic learning ^33^.

### G. Unintended effects

The interviews explicitly asked about unintended negative effects. None were noted; however, some risks were highlighted, especially that of overloading CHWs. There was also concern about working with ‘unfunded’ CHWs – and the potential around expectations for getting on payroll - and for that reason, after cycle 3, VAPAR only trained in areas with funded CHWs. Raised expectations is always a risk for research projects which do not bring substantial resources into an area, however, the team managed this carefully. Some positive unintended effects have already been highlighted, but there were also others, such as community members gaining employment based on their increased skills and confidence (KI 17). The Scottish application of the method, which led to practice change in a 12-week pilot, was similarly unanticipated.

### H. Underlying assumptions

Some underlying assumptions were identified during the development of the theory of change, some of which were themselves potentially influenced by the programme, such as:

•   the research institutions, DoH and local communities being able and willing to engage over time and being open to dialogue;

•   the three core constituencies (communities, health system and researchers) having some flexibility of resources to be able to respond to new co-produced evidence;

•   there being sufficient social coherence to support movement towards shared priorities and actions;

•   there being sufficient stability in the health sector for receptivity to programme outputs;

•   there being a wider interest in distributed and collaborative PAR (in relation to transfer of lessons to other settings).

These have held to some extent but remain ambitious in a context where flexibility of resources is a particular challenge ^19^ and there are many social divisions, which still present challenges for sustainability, impact and scaling. Stability in staffing on the public service side has however been good over the programme period. With hindsight, there are assumptions which we might add now, as these have proved challenging, but were not foreseen. These include:

-   absence of shocks (though COVID-19 did in fact prompt a useful pivot in the VAPAR model)

-   continuity of research staff (the team has faced quite a lot of turnover due to a variety of factors) and

-   supportive research environments (staff have shown considerable commitment while facing challenging workloads and academic environments).

### I. Supporting resources

It is important to set the resources invested against programme achievements, also to guide future groups. VAPAR had a budget of £900,000 across six years, including an additional £200,000 raised from a variety of small grants after the initial HSRI grant. Although the number of people involved in the programme was large, in full-time equivalent posts, the original budget supported the inputs of eight co-investigators and equated to 2.5 full-time staff (made up of part-time engagement by a range of senior, mid-level and junior researchers) on average over the lifetime of the grant. The outputs are large in relation but more broadly, value for money is hard to judge, given the importance of intangible outcomes (such as trust, skills and collaborative relationships), the length of time needed for these to bear fruit (and their uncertain duration) and the complexity of assessing programme contribution in dynamic environments.

### J. Recommendations

KIs made a range of recommendations. In relation to the current VAPAR model, local participants requested continued support, a continued focus on CHWs, nurses and community members, and scaling up in the province. One KI recommended a more consistent engagement of higher-level managers. There were suggestions that a consistent thematic focus would help deepen relationships and understanding of the topic and feed into clinical practice, linked to DoH priorities. In terms of sharing lessons and practice, continued reflection, documentation and sharing of experiences was encouraged, including working with practitioners in the region and beyond who are struggling with similar challenges, using peer learning and practitioner networks and testing the model in new contexts to enhance generalisability of findings. Several KIs recommended simplifying communication of the approach. To sustain the work, it was recommended that VAPAR make the model more standardised, move away from *ad hoc* structures, and link with wider supportive structures and legislation, and support the community to participate more effectively in critical reflections on the work. In relation to the research ecosystem, it was recommended that the programme continue to work to try to align researcher incentives to support this embedded and participatory approach (e.g. engagement as a mandatory component from research ethics perspective, recognition for impact and resourcing for it, collaborative working structures), but also that it was necessary to push back on unrealistic expectations of short-term impact from funders.

## Discussion and conclusion

VAPAR had ambitious goals, including adapting to learning and a changing environment. It had many successes. For those with whom it worked directly – at multiple levels of the system – it has been able to create spaces for respectful and inclusive dialogue, despite complex hierarchies as well as improve the relevance, quality, and uptake of research data. There is evidence of growth in skills, confidence, motivation and agency, especially amongst CHWs, and a greater clarity on and recognition of their roles from health staff, communities and CHWs. It brokered trust relationships between communities and the authorities, resulting in new commitments to, and capabilities in, evidence-informed decision-making. CHWs developed new community mobilisation and analytical capabilities; and reported improved community-acceptance; peer support; and recognition by the system. The relevance of community voice has been raised, and capacity built at different levels, including provincial, to support and enforce more engaged and potentially productive community health research. Relationships have been built between levels of the health system, as well as with wider stakeholders from other sectors. Some gains are documented in terms of health behaviours and health service provision, with the potential, if sustained, for longer term impacts on health outcomes and equity. At the same time, there has been considerable investment in sharing tools and lessons within the province and country and internationally through a range of products and approaches, with evidence of uptake across Mpumalanga, and in other settings.

Reflecting on the theory of change, inputs were broadly delivered as anticipated, and all mechanisms were triggered, along with some unanticipated ones, such as role clarification. Outputs were all achieved to some extent, while outcomes varied, as is common: the further along the theory of change, the harder it is to demonstrate contribution. We note that the domains are dynamic and interconnected, with causality likely to flow backwards as well as forwards, and there is also no necessary hierarchy. While outcomes are important, they are not exclusively so and can be achieved in the short term without longer-term benefits which, say, increased trusting relationships might deliver. These ‘software’ aspects are key to strong and resilient health systems, as articulated recently in a health system strengthening evaluation collaborative ^37^. We argue that learning platforms can deliver intrinsic *and* instrumental benefits, and the former should be recognised.

The key underlying assumption of VAPAR - that practical, experiential knowledge co-constructed, self-reflective and embedded in complex, adaptive social and health systems will support and inform the organisation and delivery of public goods that are equity-oriented and people-centred ^1^ – benefits from a supportive context. Our work on decentralised decision space, social accountability, and community power-building, shifted the focus to the contexts and environments of learning as well as learning processes, informing broader shifts towards ‘state-society synergies’. Nevertheless, the health sector context remains challenging in terms of resourcing, political pressures and organisational culture in particular, as supported by wider literature in South Africa ^19^. Nevertheless, that can support and enable learning as a routine process. Otherwise, changes related to funding stability as well as epistemic factors are needed in the research ecosystem.

The PAR component emerged strongly, supporting wider evidence that communities can address problems within their own resources. Our experience also supports the conclusions that ‘community engagement is neither easy nor cheap and requires a great deal of technical competency and resources. It is a dynamic and demanding process that needs appropriate support; but the pay-offs in health and society can be immense when done correctly’ ^38^. In corroboration, a recent study found that the best-performing health facilities were characterised by good management and leaders who could engage staff and community members ^39^. Primary health care depends on empowered and engaged communities, but testing different approaches to engagement is still required and VAPAR has added to this important body of literature. We have also highlighted the potential of CHWs as PAR agents ^14^, alongside the support that is needed to enable them to carry out these crucial roles in an empowered (as opposed to exploited) manner.

Perspectives brought to the evaluative reflections by stakeholders are noteworthy. Some had a more pragmatic approach to the VAPAR model, arguing for a less community-led prioritisation of topics in favour of more targeted, sustained and actionable health topics of known interest to the DoH. Others argued for going even further in relation to community empowerment at all stages of the cycle and regretted a diminution in the role of the community in the later cycles. Equally, there were trade-offs between the focus on bringing in marginalised voices and ensuring that some leaders with power to take actions were represented. These are dilemmas which have been experienced in other health system learning platforms ^34^. Managing power dynamics and the ‘locus of control’ is challenging in such collaborations. Within the research team, there was a shift in leadership from north to south over the life of the collaboration, as planned and expedited by COVID-19, which prevented northern researchers from travelling. However, it is hard to achieve full parity of decision-making, with power often remaining with external actors e.g. funders. 

Some enablers emerged as supporting the areas where achievements were made. These included the commitment of the team members, who showed high engagement, mutual support and entrepreneurship in seeking and taking advantage of opportunities and accessing additional funds. The long span of the grant – six years, building on an 18-month development grant –allowed for relationships and capacity to be built and for the model to be reiterated. The MRC/Wits Agincourt HDSS provided a stable platform, with a strong community engagement record, and there were many committed individuals within the local health system, who engaged as allies. The steering committee, which included local health system representatives and academic thought-leaders in South Africa, the region and internationally, provided excellent guidance, and some research team members developed effective PAR skills. As ever, individuals and relationships matter.

Several unintended positive impacts emerged, including South to North learning, strengthening of the Provincial Health Research Committee, and indeed many of the materials, such as the CHW manual. Overall productivity was high for small core team of 4-5 researchers; however, this reflects productivity pressures and norms in higher education, and, ironically, a lack of time for reflection was reported due to the high pace of activities, especially towards the end of the programme. As well as enablers, there were barriers to effectiveness, which include lack of functioning representative structures in the local health system, which could have been strong partners for VAPAR (such as health clinic committees) ^40^. A change in DoH provincial staffing early in the programme also reduced their engagement as active partners in VAPAR, as originally intended.

We note limitations in relation to this study, especially the challenge of evaluating a ‘moving target’, given the emergent programme design, but also the complex nature of the intervention and the importance of ‘software’ changes that are hard to precisely measure. Most of our data is qualitative and perceptual, but perceptions are real and matter, so our focus has been on understanding and explaining them.

In terms of positionality, the team was primarily South African, based at the MRC/Wits Agincourt HDSS or Mpumalanga Department of Health and in the UK affiliated to the MRC/Wits Agincourt HDSS. The team have a long-standing collaboration with a shared commitment to evidence-informed learning in health systems, and embedded research. The PAR framework encouraged regular reflection and critique, including on tensions between a time-limited research project and objectives related to sustainability. This led to the leveraging of additional support for continued activities and informed close working with the provincial DoH to ensure practical relevance. Within the team, positionality issues were considered, such as who controls funding, collects, and analyses data, who publishes, and whose perspectives are prioritised. This was informed and supported by the MRC/Wits Agincourt HDSS, a centre grounded in commitment to rural communities over decades.

Developing a learning platform is ambitious and we highlight some of the tensions and trade-offs involved, including challenges in relation to sustainability. However, the evaluation does support the original programme proposition that bringing empowered community voices into health system planning and decision-making is feasible and impactful and emergent through collective, pragmatic, and adaptive processes. This supports the argument that learning health systems are a core, but neglected, component, especially in LMICs, and one which should receive more attention and resourcing ^41^. Our participatory learning site linked information with deliberation and action, as highlighted in the Alliance Flagship report on Learning Health Systems. This article adds to the limited published evaluative evidence on such platforms in LMICs.

## Ethics and consent

The study protocols for Cycles 1-3 were approved by the University of Aberdeen (Cycle 1 2017-18 UOA CERB/2019/1/1693, 11/10/2017; Cycles 2-3 2019-21 CERB/2019/1/1693 23/01/2019) and the local review board at Wits (Cycle 1 2017-18 M1704115 01/12/2017; Cycles 2-3 2019-21 M1811109, 18/01/2019). An amendment was approved for the final evaluation by the local review board (Evaluation 2022 Amendment to Wits M1811109, 09/09/2022).

As part of these processes, research involving human participants must adhere to the Declaration of Helsinki. Written informed consent was gained from all participants in the study. Participants were informed about the nature of the programme and its evaluation, its aims, objectives, procedures and outcomes. Participants were assured that identifying information would be anonymised and would not be disclosed beyond the research team without permission. All participants were free to leave the study at any time and for any reason. Efforts to develop partnerships and processes beyond the programme were sought throughout both the programme and evaluation.

## Data Availability

Most of the data supporting this article are contained in the article, its tables and supporting references, and the VAPAR website. The transcripts for the final evaluation interviews contain material which could identify individuals, their views and positions and sharing would therefore violate the confidentiality agreement. Applications for this data can be made to the lead author by email ( switter@qmu.ac.uk) but would only be considered if transcripts could be heavily redacted to protect identities.
