# Community and health systems learning: critical realist evaluation of the VAPAR ‘learning platform’ in rural South Africa 2015-25

**DOI:** 10.12688/wellcomeopenres.23381.2

**Published:** 2025-12-12

**Authors:** Sophie Witter, Lucia D'Ambruoso, Maria van der Merwe, Jennifer Hove, Nombuyiselo Nkalanga, Denny Mabetha, Gerhard Goosen, Jerry Sigudla, Stephen Tollman, Verbal Autopsy with Participatory Action Research (VAPAR)/Wits/Mpumalanga Department of Health Learning Platform

**Affiliations:** 1 Institute for Global Health and Development, Queen Margaret University, Edinburgh, Musselburgh, Scotland, UK; 2 Aberdeen Centre for Health Data Science, Institute of Applied Health Sciences, School of Medicine, Medical Sciences and Nutrition, University of Aberdeen, Aberdeen, Scotland, UK; 3 MRC/Wits Rural Public Health and Health Transitions Research Unit (Agincourt), School of Public Health, University of the Witwatersrand, Johannesburg, Gauteng, South Africa; 4 National Health Service (NHS) Grampian, Aberdeen, Grampian region, UK; 5 Department for Epidemiology and Global Health, Umeå University, Umeå, Västerbotten County, Sweden; 6 Department of Global Health, Stellenbosch University, Stellenbosch, Western Cape, South Africa; 7 MV Consulting, White River, South Africa; 8 Mpumalanga Department of Health, Mbombela, South Africa

**Keywords:** learning platform; community health; health system strengthening; learning health systems; South Africa; participatory action research; theory-based evaluation

## Abstract

**Background:**

Learning platforms can strengthen primary healthcare (PHC) by integrating community knowledge with system decision-making, but evidence on how they work in low-resource settings is limited. This study presents a realist evaluation of the Verbal Autopsy with Participatory Action Research (VAPAR) learning platform in rural Mpumalanga, South Africa (2015–25). VAPAR aimed to embed participatory evidence generation and shared learning within routine district processes to support more equitable, community-linked PHC.

**Methods:**

A realist design was used to synthesise data from five action-learning cycles (2017–23), a preceding pilot (2015–16), and an engagement and uptake phase (2023–25). Data included cycle reports, participatory outputs, verbal autopsy (VA) analyses, 22 endline interviews, policy, strategy and planning documents. Using a co-developed theory of change, qualitative data were coded to examine context-mechanism-outcome patterns. Mechanisms were identified and refined through cross-cycle comparison, triangulation, and stakeholder validation.

**Results:**

VAPAR was contextually responsive, adapting to shocks such as COVID-19 and progressively embedding within the district health system. Through regular dialogue, the platform activated generative mechanisms of trust-building, role clarity and recognition, collective sense-making, and strengthened agency, particularly among Community Health Workers (CHWs), whose skills, confidence and legitimacy expanded. These mechanisms operated within an enabling structural context shaped by PHC reforms that strengthened the District Health System and Ward-Based Primary Health Care Outreach Teams, alongside trade-union action for CHW absorption into public service. Institutionalisation followed through Mpumalanga’s revitalised Health Promotion Programme, with adaptation to additional provinces and for outbreak response and emergency obstetric care. Outcomes were interpreted through context-mechanism-outcome patterns, illustrating how participatory learning becomes embedded in decentralised health systems.

**Conclusions:**

Over a decade, VAPAR demonstrated how structured, participatory learning can reshape relationships, strengthen community-linked PHC, and support institutionalisation of routine, evidence-informed practice in decentralised health systems. The findings offer transferable lessons for sustaining learning platforms in resource-constrained settings.

## Plain language summary

This study evaluated the Verbal Autopsy with Participatory Action Research (VAPAR) learning platform in Mpumalanga Province, South Africa. VAPAR was developed to support generation and use of evidence in the health system by involving communities directly in decisions about health priorities and services. The evaluation covered the full, funded programme (2015–25) and used a theory-based approach combining data from five reflection and action cycles, together with pre- and post- phases. It explored how locally produced evidence and shared learning affected collaboration, demand for data, and the quality of health services.

VAPAR adapted to major challenges such as the COVID-19 pandemic and created spaces for open, respectful dialogue between communities, health workers, and managers. The process built confidence and practical skills among Community Health Workers (CHWs) in problem-solving, analysis, and peer support. Their role in the health system became better recognised, which helped to build trust between communities and authorities and encouraged more evidence-based decision-making. Some CHWs trained through the programme were later absorbed into government positions, and VAPAR’s participatory training model is now being expanded within and outside the province and integrated into Mpumalanga’s revitalised Health Promotion Programme.

Sustaining such a platform requires ongoing support and coordination. Nevertheless, the experience shows that learning platforms like VAPAR can strengthen relationships, accountability, and equity in healthcare. The study also highlights the difficulty of measuring change in complex systems but demonstrates how collaboration between communities and health systems can build resilience and lasting capacity for improvement. When people are given the means and permission to reflect together on evidence that matters, they begin to act differently and, in doing so, reshape the institutions around them.

Word count (main body): 7,287

## Introduction

Learning Health Systems (LHS) have gained prominence as a means of improving primary healthcare (PHC) through structured cycles of evidence generation, reflection, action, and continuous learning ^1– 3^. However, in low- and middle-income countries (LMICs), where institutional fragility, resource constraints, and entrenched inequities shape service delivery, there remains limited empirical understanding of how such learning processes can be built, sustained, and institutionalised ^4– 6^. This paper presents an evaluation of the 10-year Verbal Autopsy with Participatory Action Research (VAPAR) learning platform in rural Mpumalanga Province, South Africa, examining how locally grounded, cooperative learning can strengthen health systems and promote equity.

### Context and rationale

South Africa’s health policy framework is grounded in constitutional commitments to the right to health and social participation, providing a favourable environment for people-centred primary health care (PHC) ^7– 11^. Substantial gains in HIV, tuberculosis (TB), and maternal and child health have been achieved through expanded antiretroviral therapy and strengthened MCH programmes ^12, 13^. However, multimorbidity and non-communicable diseases (NCDs) now contribute more than half of national mortality, with the highest burden among the poorest quintiles ^14^. This has placed renewed emphasis on continuity of care, health promotion, intersectoral collaboration, and strengthened district health systems ^9, 15, 16^.

These priorities respond to and align with ongoing reforms. Through National Health Insurance (NHI), the District Health System (DHS) revitalisation strategy, and the PHC Re-engineering strategy, Community Health Workers (CHWs) are recognised as key connectors between households and the health system. Despite recognition in policy and strategy, in practice CHWs often face precarious employment, uneven supervision, and limited opportunities for skill development ^17, 18^. Strengthening analytical, and relational capabilities is therefore important as a PHC implementation strategy support to realising South Africa’s health sector ambitions.

VAPAR is based in the rural province of Mpumalanga, a largely rural province of 5 million in northeastern South Africa ^19^. Mpumalanga is one of South Africa’s poorest provinces. The province faces persistent burdens of HIV and injury, rising multimorbidity, limited operational decision-space, weak integration between community and facility actors, and historically low use of local data for decision-making ^19– 23^. These conditions underscore the need for grounded, collaborative learning processes that enable communities and health workers to learn together from locally generated evidence integrating community knowledge with system priorities.

### The VAPAR programme 2015–25

VAPAR was established in 2015 as a partnership between the Mpumalanga Department of Health (MDoH), the MRC/Wits Agincourt Health and Socio-Demographic Surveillance System (HDSS), and academic collaborators. It sought to embed a system of knowledge production and exchange linking routine Verbal Autopsy (VA) with community-based Participatory Action Research (PAR). A 2015–16 pilot developed the Circumstances of Mortality Categories (COMCAT) classification ^24, 25^ and tested community-led deliberation of locally relevant mortality evidence ^26– 31^. Five reiterative action-learning cycles (2017–23) integrated VA data with participatory problem analysis and collective planning, action, and reflection ^31– 36^. From 2023–25, the approach was adapted and scaled across all three health districts in Mpumalanga and shared with partners nationally and internationally through the South African Population Research Infrastructure Network (SAPRIN) ^37^.

### Theoretical and conceptual framing

The evaluation began with a Realist Evaluation (RE) design, using a co-developed Theory of Change (ToC) as an initial programme theory (middle range theory) of how VAPAR worked, for whom, and under what conditions ^38^. As the programme progressed and matured, emerging changes particularly in relationships and institutionalisation, were observed. In response for this evaluation, we extended the analytic frame using Critical Realism (CR) to deepen explanatory power. Unlike traditional analyses that summarise evidence, CR identifies underlying causal mechanisms that influence outcomes and explains how these mechanisms operate in particular contexts. It begins with one or more candidate theories about how interventions work and uses evidence to test and refine them. The goal is to move from surface-level correlations to explanatory understanding.

CR enabled us to: identify underlying generative mechanisms shaping behaviour and interaction; examine how structural conditions (e.g., labour arrangements, policy reforms, organisational hierarchies) enabled or constrained these mechanisms; trace emergent system changes across cycles (including uptake and changes in behaviours and services); and theorise morphogenetic processes through which interactions reshaped roles, relationships, and routines ^39– 43^. The combined approach supported an analysis that moved beyond describing programme activities to explain which generative mechanisms were activated over time, how and why, and under what contextual conditions they produced system level effects, viewing health systems as open, adaptive social systems shaped by structures, agency, and context ^44, 45^.

### Aim and contribution

This paper evaluates whether, how, and why the VAPAR learning platform contributed to health system strengthening in Mpumalanga over a decade of implementation. Specifically, we aim to:

1. Describe how the programme evolved in relation to its context and stakeholders;
2. Identify generative mechanisms linking participatory learning to changes in relationships, practices, and capabilities in communities and the health system;
3. Examine outcomes relating to service processes, data use, and institutionalisation; and
4. Draw transferable lessons for sustaining learning platforms in decentralised health systems.

By integrating a decade of evidence through a critical realist explanatory lens, this evaluation contributes to scarce empirical literature on learning health systems in LMICs and offers practical and conceptual insights into how community-linked learning can be embedded and sustained in real-world systems undergoing epidemiological and policy transition.

## Methods

### Design and analytical perspective

The evaluation was grounded in a realist understanding of change as produced by underlying mechanisms in social relations, organisational norms, and material conditions, activated through actors’ reasoning and responses in particular contexts ^46, 47^. The CR stance framed health systems as open, complex, layered, and adaptive ^48, 49^, enabling us to theorise how VAPAR contributed to change across multiple cycles and institutional levels. Using the pre-existing ToC, we combined RE logic with CR reasoning to examine how outcomes emerged from interactions between actors, structures, and evidence; and to build an explanatory account of cooperative learning within an evolving health system. The participatory orientation ensured that both data and interpretation were co-produced with stakeholders, reflecting the principle that learning in complex systems depends on dialogue and the integration of diverse forms of knowledge ^50, 51^.

### Scope

The evaluation covered the full trajectory of VAPAR: a pilot phase (2015–16) with integration of VA and PAR, development of COMCAT, and early testing of community-led deliberation across a range of conditions ^24^; the main programme phase (2017–23): five iterative learning-and-action cycles involving engagement, analysis, action, and reflection among communities, CHWs, local officials and health system actors; and post-programme uptake and expansion (2023–25): adaptation and scale across Mpumalanga’s three health districts (Ehlanzeni, Gert Sibande, Nkangala), diffusion through the SAPRIN, and application to national and provincial priorities. Together these provide a ten-year window through which to trace how mechanisms of learning and collaboration were initiated, sustained, and institutionalised.

### Theory of change

**Figure 1.**
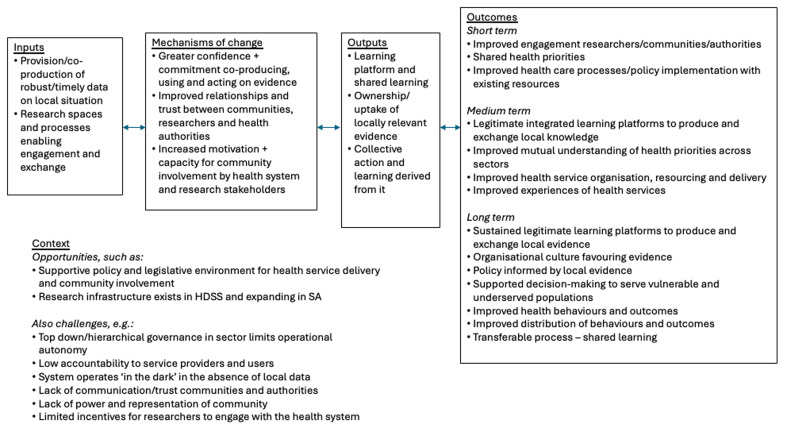
Initial VAPAR theory of change. (This figure has been reproduced with permission from Source: 32 under a Creative Commons Attribution 4.0 International License, which permits use, sharing, adaptation, distribution and reproduction in any medium or format).

The ToC was co-developed with community, health system, and research partners and revised in reflection workshops ( Figure 1). The initial theory proposed that if locally relevant evidence was co-produced in inclusive, action-oriented deliberative spaces, then relationships would strengthen, leading to improved problem-solving and, ultimately, more equitable PHC delivery ^38^. The ToC guided data collection and analysis across five domains: context, inputs, mechanisms, outputs, outcomes. As the programme matured, new feedback loops were added, particularly around CHW empowerment, peer-learning, and policy uptake ^32^. The initial ToC was a logic-based formulation of expected pathways. In this evaluation, it was refined analytically, positioning mechanisms as causal links between programme activities and outcomes. This is consistent with RE and was extended through CR reasoning on emergence and structural conditioning.

### Sources

Data were drawn from diverse sources across community, facility, district, provincial, and research actors ( Table 1). Internal programme documentation included protocols, cycle reports, reflective summaries, workshop notes, and 29 published or accepted papers. Participatory materials: photovoice, problem-tree diagrams, Venn maps, action plans, and cycle reflections provided insights into collective reasoning and change processes. VA datasets from the Agincourt HDSS (2015–22), including COMCAT, were also analysed to explore trends in mortality and care pathways. Qualitative evidence was central, reflecting the programme’s focus on relationships, trust, and practice. End-of-cycle key-informant interviews and stakeholder workshops captured changes in roles, interactions, and decision-spaces. Pre- and post-training interviews were conducted with CHWs in cycles 3–5. For the 2023–25 uptake phase, sources included Department of Health correspondence, documentation of scale-up activities, provincial/district performance reports, budgets, and staffing data to assess resource intensity and value for money. Twenty-two endline interviews were conducted with programme stakeholders.

**Table 1.** Summary of data sources for evaluation.

Data sources	Description
	*Internal to VAPAR*
VA and PAR outputs	E.g. analyses of VA data, Photovoice images, root cause mapping using problem tree, Venn diagrams, action pathways produced by community participants across five VAPAR cycles
Cycle reflections and evaluations	Published for cycles 1, 2 and 3; and evaluation papers on cycles 4 and 5 are forthcoming (reports on these cycles are used instead in this evaluation)
Published papers	29 VAPAR research papers published to date; these are referenced on relevant topics
Evidence briefs	13 produced in English and Tsonga, feeding evidence on chosen priority topics to community and system stakeholders
Local action plans and follow up	Produced in each cycle in relation to action on alcohol and drugs, access to water, and reducing loss to follow-up for TB and HIV/AIDS treatment
Final interviews	22 final key informant interviews conducted for the final evaluation using a semi-structured topic guide. Participants were chosen for their knowledge of the programme and included community partners (3), health system partners in the district and province (5), researchers (9), national collaborators (2), and international collaborators and advisers (3). Of the 22, 14 were female and 8 male.
Programme expenditure	Based on programme budget and further grants; assessed to inform on resource intensity of VAPAR
Stakeholder mapping	Conducted by research team at the end of each cycle, mapping key stakeholders against influence over and interest in the focal topic
VAPAR workshop reports	Each stage of each cycle included workshops with diverse participants; their feedback and team reflections.
Other team outputs	Public engagement writing (e.g. The Conversation pieces); podcasts by team; short videos; training manual; research ethics training for province; local radio series in local languages
	*External to VAPAR*
Official documents from the health sector	For example, the District Health Plan, Annual Performance Plan, Integrated Development Plans, quarterly and annual reviews
National/sub-national policy and strategy documents	Relevant policies affecting community health, such as the National Health Insurance (NHI) plans, PHC Re-engineering strategy, and policies relating to relevant other sectors, such as water, alcohol and drugs)
MRC/Wits Agincourt Unit data and research	Other ongoing studies and outputs of relevance (e.g. data on clinic functionality)

### Sampling and data collection

Endline participants were purposively selected for their experience and knowledge of the programme, spanning community, facility, sub-district, district, provincial, and research-institutional levels. Recruitment was facilitated through established engagement structures within the Agincourt HDSS and authorised by health system managers. Sampling aimed for gender balance and inclusion of both paid and unpaid CHWs. Data collection was undertaken by trained researchers fluent in Xitsonga and English. All interviews were recorded (with permission) and transcribed verbatim.

### Data analysis

Analysis combined framework analysis with realist analytical principles ^52, 53^. All qualitative data were imported into NVivo 12 and coded using the ToC domains. For each domain, we examined patterns linking contextual features with actors’ reasoning, interactions, and reported outcomes. Candidate generative mechanisms were identified and tested across actor groups, cycles and sites linking mechanisms to outcomes specifying contextual feature that enabled or constrained them.

Quantitative elements, including mortality trends with VA computation using the InterVA-5 probabilistic algorithm ^54^, and programme participation statistics, were triangulated with qualitative insights and through iterative validation with stakeholders through 2023–24 reflection and synthesis workshops. Retroduction was used pragmatically to infer the underlying properties or capacities necessary for observed patterns to occur. This iterative process of co-interpretation was empirically and methodologically and consistent with the programme’s learning orientation ^55^.

### Limitations and reflexivity

Given the research team’s role in programme implementation, reflexivity was essential. Team members maintained reflective journals and analytic memos. An independent advisory group of senior academics and provincial officials periodically reviewed emerging analyses to challenge assumptions and preserve analytic distance. Causal inference was supported through triangulation, replication of context-mechanism-outcome (CMO) patterns across cycles, and corroboration from diverse stakeholder groups. While primarily qualitative, the approach drew on the realist principle of analytic generalisation: explaining how change occurs under specific conditions to elicit lessons for other contexts ^25^.

### Ethical considerations

Ethical approval was granted by the University of the Witwatersrand Human Research Ethics Committee (M170846) and the University of Aberdeen College Ethics Review Board (CERB/2022/10/04). Renewed clearance for the final synthesis was obtained in 2022. All participants provided written informed consent. Confidentiality was prioritised given the small and interconnected communities involved. The study adhered to the Declaration of Helsinki (2013) and South African regulations governing research with human participants.

## Results

### 1. Programme trajectory and contextual conditions

Over ten years, VAPAR evolved from a pilot linking VA with PAR into a district-embedded learning platform aligned with PHC governance. The progamme adapted and evolved in response to contextual shocks, organisational change, and shifting systems priorities. The 2015–16 pilot introduced the combined VA-PAR approach, tested community-led deliberation of mortality evidence, and developed the Circumstances of Mortality Categories (COMCAT) tool. Building on this foundation, five reflection-and-action cycles (2017–23) progressively shifted from community-nominated priorities in a limited geographical area to a CHW-centred, district-linked learning platform embedded into routine PHC governance ( Table 2).

**Table 2.** Summary of VAPAR cycles.

Cycle and duration	Participants	Focal theme	Location/coverage	Summary of process	Key adaptation/ contribution	References
1 (2017–19)	48 community members	Access to safe water; alcohol and other drugs	3 villages in Agincourt DHSS area	Multisectoral action plans developed and implemented with departments of Water and Sanitation, Basic Education, Social Development, and : South African National Council on Alcoholism and Drug Dependence (SANCA)	Established foundational PAR cycle and community-led action plans	Cycle 1 reports ^26, 28– 31, 33– 35, 57^
2 (2019-20)	54 community members	Continuation of above, with co-redesign at onset of COVID-19	3 villages in Agincourt DHSS area	Interrupted by pandemic; co-redesigned to support district response, leading to focus on CHWs and HIV/TB	Pivoted model to support pandemic response and identify CHWs as key agents	Cycle 2 reports ^36^
3 (Feb-Apr 2021)	9 CHWs, 6 community mentors, 27 community members	Loss to follow-up (LFU) for TB and HIV/AIDS	3 villages in Agincourt DHSS area	Developed and piloted rapid PAR training for CHWs through 16 workshops	Introduced CHW-centred participatory training and mentorship	Cycle 3 reports ^32, 37, 58^
4 (Mar-May 2022)	54 CHWs, OTLs, 3 CHW mentors (from C3), 53 community stakeholders	LFU for TB and HIV/AIDS	6 local areas in Bushbuckridge sub-district	Delivered 41 workshops alternating theory and practice using revised training manual	Expanded to district level; established CHW peer-support and leadership model	Cycle 4 reports
5 (Feb-May 2023)	53 CHWs, 10 CHW mentors, 52 community representatives	LFU for TB and HIV/AIDS	Bushbuckridge sub-district, 5 additional local areas	Delivered 8–10-week “training-of-trainers” programme, one 2–3-hour workshop per week	Institutionalised CHW-led training; positioned for provincial scale-up	Cycle 5 reports

Early cycles focused on water access and substance use, producing multisectoral action plans. These were valued for community ownership but revealed weak alignment with health system priorities, prompting adaptation and closer integration with District Health Management Team (DHMT) structures. Cycle 2 (2019–20) was interrupted by COVID-19, but the crisis catalysed a co-redesign with communities and district authorities centred on CHWs as first-line responders. This strategic pivot aligned the platform with core district system priorities.

Cycles 3–5 (2021–23) consolidated the CHW-centred model. A rapid PAR training package evolved into a training-of-trainers (ToT) approach, delivered across multiple local sites, with workshops alternating between theory and practice. By 2022, a peer-led CHW learning forum had been established, creating regular spaces for reflection and problem-solving beyond the researcher-led cycles. By Cycle 5, VAPAR was operating across most of Bushbuckridge sub-district and interfacing routinely with Ward-Based Primary Health Care Outreach Teams (WBPHCOTs).

*CHWs are key to government policy now… so the current VAPAR model is spot on (KI15)*

The programme’s evolution occurred within a system characterised by hierarchical authority, limited operational decision-space, resource constraints, and weak integration between facility and community actors. Informants described a system “operating in the dark” without evidence or data and limited representation of community perspectives. The challanges of confronting entrenched divisions between service users and providers, and the political nature of operational decision-making were also highlighted.

*Are we really able to confront underlying system constraints, for example--- the culture of blaming patients? (KI6)*
*----operational issues still have to follow politics (KI15)*

Despite challenges, policy reforms and governance contexts created opportunities. PHC re-engineering and NHI debates re-focused attention on the district health system, PHC and community participation; and structures such as the Provincial Health Research Committee and SAPRIN offered institutional platforms for evidence generation and use. Local governance cultures where interpersonal relationships mediated influence, and cross-level collaboration was rare ^56^, also meant that VAPAR with its focus on relationships and cooperative problem-solving was well placed to bridge gaps between communities, frontline providers, and management.

Evidence briefs synthesised statistical and experiential data for community members, CHWs, and managers in accessible formats and languages. These supported shared interpretation of locally relevant evidence. While beneficial in terms of regular spaces to think and act differently, early experience highlighted the need for health sector alignment. In cycle 1, the focus on community-nominated priorities of water, alcohol and drugs, while community-owned, presented challenges for MDoH stakeholders, whose statutory mandate did not extend into social determinants.

The COVID-19 co-redesign created strategic alignment between community priorities and system mandates. Subsequent cycles focused on TB/HIV treatment retention, CHW roles, and outreach team functioning. These were directly linked to both community needs and district and provincial priorities such as WBPHCOTs, the Medium-Term Strategic Framework, the National Development Plan (NDP) Implementation Plan 2019–24, and the MDoH strategic plan 2020–24. The provincial Annual Performance Plan (APP) and district health plan (DHP) emphasised quality of care and CHW competencies in community mobilisation ^59^.

As VAPAR came to be seen as supportive of system priorities, rather than external to them, and institutional traction strengthened and relationships stabilised. From here, ideas about shared ownership were possible to expand. Stakeholders frequently described VAPAR as distinctive because of its embedded participatory processes within formal system structures:

*It was unique in that it took participatory research into an institutional environment (KI 6)*

Post COVID, intensified trade-union action for CHW absorption into public service raised the cadre’s visibility. Increasing demand from district managers enabled extension beyond the surveillance area, linking with WBPHCOTs, sharing with other provinces and SAPRIN nodes. By 2023–25, the approach was adapted across Mpumalanga’s three districts and diffused to other provinces through SAPRIN. This trajectory provides the context through which subsequent mechanisms were triggered and outcomes generated.

### 2. Mechanisms

The ToC proposed that co-produced evidence discussed in inclusive deliberative spaces would strengthen relationships and problem-solving, leading to more equitable PHC. Across data sources, four generative mechanisms consistently explained how this occurred: (i) trust-building; (ii) role clarification and recognition; (iii) collective sense-making; and (iv) empowerment/agency. The mechanisms interacted and were shaped by contextual conditions.

***2.1 Trust-building***. Regular, facilitated encounters between communities, CHWs and health officials created relational stability in a system marked by fragmentation and hierarchy. Participants described a shift from blame to constructive dialogue, enabling frank discussions and a collective willingness to act. Trust was seen as fragile and contingent: “a municipal manager coming to the meeting and being honest --- doesn’t equate to generalised increase in trust” (KI 7). Nonetheless, researchers’ relative neutrality and consistent presence created a sense of predictability, credibility and safety, particularly for CHWs and community members, in spaces where it was possible to think and act differently:

*I was impressed ---many people were present---Community members, traditional authorities, and other departments--- Other researchers come, collect data and go---this one was very different (KI 3)*
*--- everyone realized that it is time to shift our ways of thinking and initiate dialogue, unite, and collaborate (Community stakeholder, reported in 35)*

***2.2 Role clarification and recognition***. CHWs’ ambiguous roles between households and clinics and between the formal system and outsourced NGOs had historically undermined confidence, legitimacy, and integration. VAPAR created spaces for CHWs to articulate their roles and challenges in front of peers and supervisors, negotiate expectations, and demonstrate competencies. Managers came to appreciate CHWs’ challenges, motivations and insights and to recognise them as integral members of PHC teams. CHWs’ growing confidence to speak in mixed forums was an important shift.

*It gave me a platform to stand in front of big managers (CHW, final workshop)*
*VAPAR taught us team building--- We are able to adapt to every community now (CHW, final workshop)*

As role clarity increased, new mutual forms of understanding and respect emerged. CHWs reported less belittling treatment in clinics, while managers described greater appreciation of CHWs’ contribution to follow-up, health promotion, and community mobilisation.

***2.3 Collective sense-making***. Joint interpretation of VA data, photovoice, and participatory tools enabled actors to deliberate over causes and responsibilities, building shared understanding of problems and options for action. CHWs reported increased confidence in analysis, and managers described a shift in staff attitudes that was more aware of and receptive to data and evidence. Providers and community members described seeing problems “with new eyes”, challenging assumptions and generating shared understandings.

*There was an increased awareness by staff of community needs--- The DoH were not previously using research data but started to realise its value (KI1)*
*People love this method--- assumptions about causes and answers are challenged (KI17)*

Most significant was the impact on CHWs. Across cycles 3–5, they reported improved skills in public speaking, problem solving, collaboration and facilitation. This was supported by observations from health managers, who reported that CHWs were spontaneously using PAR tools in routine practice. Problem-tree analysis was repeatedly identified as a useful tool. Collective sense-making thus translated shared analysis into practical commitments.

*Now I know how to approach a problem, using the problem tree (CHW, final workshop)*
*In VAPAR, everyone is encouraged to participate and contribute to decision-making. This made CHWs understand their value and that together with other stakeholders, they could make a difference (KI 21)*

***2.4 Empowerment and agency***. As trust deepened and roles clarified, confidence to act and a sense of ownership grew as participants, primarily CHWs but also community partners, experienced small wins though the implementation and review of local action plans. This encouraged greater initiative and peer leadership, particularly among CHWs.

*They [CHWs]--- know their patients very well--- They have the power to change things (KI16)*
*The community learned how to problem solve--- They are more likely to talk to providers--- rather than protesting (KI17)*

Over successive cycles, agency became more collective. CHWs trained in earlier cycles acted as mentors in later ones, and community representatives increasingly led discussions and action planning. Empowerment thus interacted recursively with trust and sense-making: empowered action reinforced credibility, which further deepened relational commitment and willingness to engage.

Table 3 summarises core context-mechanism-outcome (CMO) configurations identified, integrating the four generative mechanisms and the contextual conditions that shaped their activation.

**Table 3.** Integrated CMO statements (VAPAR causal pathways).

Context (C)	Mechanism (M)	Outcome (O)
Fragmentation and hierarchy: health system characterised by habitual blame and lack of trust between facility staff, CHWs, and community members.	Trust-building: VAPAR provided regular, neutral, facilitated safe spaces where priorities including conflicts were discussed, enabling mutual respect and credible relationships.	Short term: Improved relational stability and mutual visibility. Increased attendance and participation in joint working forums.
Data under-utilisation: Low use of evidence and data for decision-making; community intelligence often unavilable, ignored or dismissed.	Collective sense-making: Processes of jointly analysing and interpreting different forms of locally-relevant and locally-owned data (VA/COMCAT) alongside PAR and community narratives (Photovoice/Problem Trees).	Short/medium term: Shared ownership of evidence; challenging of existing mental models and assumptions; formulation of highly feasible and focusssed action plans
CHW marginalisation: CHWs face ambiguous roles, precarity, and belittling treatment in clinics and communities.	Role clarification and recognition: Providing a formal platform for CHWs to lead learning dialogues, negotiate roles, demonstrate competence, and be formally acknowledged by PHC managers, clinic and outreach colleagues, community members and peers.	Medium term: Increased service activites including resource coordination between community and facility actors. Managers begin treating CHWs as integral health system members.
Alignment and structure: The programme was long-term (10 years) and strategically aligned with existing policies: PHC Re-engineering.	Empowerment and agency: The social energy generated by regular dialogue, leanring, mutual recognition and trust, enabled actors to proactively take initiative and innovate.	Medium/long term: Proactive CHW agency demonstrated in tasks (e.g., lost-to follow up tracking, COVID-19 response). Institutionalisation of VAPAR processes into routine DHS planning.

### 3. Outputs

The ToC anticipated three main outputs: (i) an established learning platform; (ii) generation and uptake of evidence; and (iii) collective action learning. By the final cycles, each was demonstrably embedded and increasingly system-owned.

***3.1 Established learning platform and quality engagement***. Engagement broadened and deepened over time. After the reiteration from cycle 1 to 2, the provincial DoH endorsed embedding VAPAR in routine PHC planning and review at district and sub-district levels, including DHMT meetings. From 2021, there was a request to roll out CHW training across the district. DHMT members, Outreach Team Leaders (OTLs), Operational Managers (OPMs), traditional authorities, clinic committees, youth groups and women’s groups all participated across cycles. As the process focused on CHW training and rapid PAR from Cycle 3, regular attendance by OTLs and OPMs was cited as evidence of institutional buy-in. Community participation remained consistently strong. By Cycle 4, workshops were largely participant-led, conducted in local languages, with CHWs facilitating analysis and action planning.

*We struggled to bring different role players into one room--- VAPAR greatly helped with that (KI 2)*
*VAPAR’s strength is in connecting different stakeholders and making them understand and appreciate each other (KI 21)*

Around 240 community participants were engaged, including from the pilot and community leaders. Over time, partners broadened participation, for example engaging youth on alcohol and drugs and women on water access. Evolving patterns of engagement, combined with co-produced evidence, alignment with PHC priorities and integration into system structures, created conditions for deeper recognition, legitimacy and shared problem-solving. The CHW peer-learning platform established in 2022 institutionalised regular reflection and mentorship beyond cycles, recognising that most CHW participants were neither fully aware of nor supported by existing outreach strategies. New spaces and platforms also emerged as CHWs were invited to speak in public fora in churches, traditional authorities and community gatherings, extending visibility and influence:

*They [CHWs] have a lot of challenges and don’t have anyone to talk to who understands their issues. There is no regular forum--- The supervisor attends and can advise, she can also bring stakeholders to the meeting, if relevant (KI 15)*
*It gave me a platform--- There was also recognition by the induna [traditional leader] (CHW, final workshop)*

***3.2 Generation and uptake of local evidence***. VAPAR’s dual evidence streams of VA data and community-generated analysis produced tangible shifts in how evidence was produced, perceived and used. Evidence was co-created for different audiences through podcasts, webinars, radio series and press articles. Managers valued accessible, locally relevant data; analysis of Agincourt VA data, for example, found that over 75% of deaths were attributable to at least one community-nominated risk factor ^57^. The programme also invested in the wider research ecosystem, supporting the Provincial Health Research Committee to develop its strategy and providing research ethics training, which contributed to the committee gaining Research Ethics Committee status and adopting a new research-uptake model. These reflected the ToC that when actors co-produce and interpret evidence, legitimacy and uptake are strengthened.

*We wanted to find a way to hold researchers accountable --- The idea came out of interaction with VAPAR--- We also realised that we needed to develop local research priorities---VAPAR helped us to think this through (KI 3)*

***3.3 Collective action learning***. Each cycle produced local action plans that were implemented and monitored. Early plans were ambitious and often dependent on higher-level authorities; later plans focused on feasible, locally controlled actions more tightly aligned with district structures and policy priorities. By Cycles 3–4, CHWs were initiating and monitoring most actions, cascading training and reflecting collectively on progress. Activities included setting up or revitalising patient support groups; health talks in clinics and during home visits; presentations at traditional authorities’ fora; and door-to-door awareness campaigns on TB, including direct observation of treatment, and support for disclosure.

*They would come up with unrealistic plans at the start but as we proceeded, they started to develop more effective and collective plans… more realistic and feasible (KI 1)*
*We not only developed action plans but also trained colleagues --- gave talks in churches --- came back together to reflect on our success and failure. --- We shared solutions across our groups (CHW, final workshop)*

CHWs also reported collaborating with the Department of Social Development, clinic committees, schools and churches, and using creative solutions such as discreet pill packaging and home gardening. The shift from ambitious to feasible actions reflects a mechanism-driven change: as actors gained analytical confidence and trust, they chose actions within their control and followed through, generating small wins that further strengthened agency. At the same time, multiple constraints were highlighted, including lack of transport, short-term employment and unreliable payment, lack of training and tools, unclear job descriptions, long distances, low remuneration, high workloads and belittling treatment in clinics:

*I know that VAPAR does not have the capacity to employ us, but if they can motivate for us or negotiate with DoH on our behalf we would really appreciate (CHW, reported in 37)*

### 4. Outcomes

Outcomes were interpreted through the ToC domains: (1) service organisation and delivery; (2) health behaviours and outcomes; (3) equitable priority setting; (4) institutionalised learning; and (5) transferable processes. Observed changes reflected context-mechanism-outcome patterns driven by strengthened relationships, role clarification and collective agency.

***4.1 Service organisation and delivery***. Informants consistently linked VAPAR to improvements in service responsiveness and intersectoral coordination. Community and health system actors described visible shifts in environmental management, enforcement of by-laws, and greater initiative from CHWs. Improvements in CHW confidence, communication and problem-solving were widely noted. During the COVID-19 vaccination rollout, Bushbuckridge’s strong performance was attributed to CHWs who were “proactive and well trained”; district managers reported adapting VAPAR practices to neighbouring sub-districts. These changes aligned with the ToC: strengthened relationships, clearer roles and regular peer learning generate improvements in service organisation and community satisfaction:

*Before VAPAR, people were throwing and dumping waste everywhere but now we have black bins---VAPAR changed the mindset of the community. (KI 20, community stakeholder)*
*As CHWs, we now use skills we got from VAPAR daily --- We are now confident enough to do our jobs ---- now the staff at the clinic understand and support us (KI 22, CHW)*
*With COVID-19 vaccination, Bushbuckridge sub-district was doing well, even national DoH wanted to know how they managed this--- CHWs were pro-active and well trained---We asked how they reached people--, they shared the VAPAR program information-– it was taken to other sub-districts (KI 10)*

***4.2 Health behaviours and outcomes.*** Population-level outcome change was not expected within the timeframe or design. However, across interviews and cycle reflections, participants described behavioural and relational shifts plausibly linked to VAPAR mechanisms. In a context of entrenched inequalities, stigma and poor relationships between services and patients, strengthened relationships and CHW confidence were associated with more responsive follow-up, earlier engagement and greater receptivity to prevention. CHWs reported greater confidence in counselling households; community stakeholders described improved willingness to engage with clinic staff.

VA and COMCAT data reflected broader contextual shocks, particularly COVID-19 — including increases in acute respiratory infection deaths from 2019 to 2021, disruptions in care-seeking, and increased use of traditional practitioners — rather than programme-specific effects. Nonetheless, participants narrated examples of behaviour change such as improved TB disclosure, better treatment adherence and greater openness to vaccination. These illustrate how social mechanisms of trust, capability and communication can create more conducive conditions for healthier practices even when structural determinants remain unchanged:

*I called and cried. I said I am crying for your life I want you to come [for TB testing]. And then he came ---We are able to have an impact --- I saved a life (CHW, final workshop)*

***4.3 Equitable priority setting***. In a system where patients were frequently dislocated from services by inequality, stigma and poor integration, regular participatory processes helped to make everyday realities of marginalised households visible to clinic staff and managers. Photovoice, problem-tree analysis and facilitated dialogue enabled CHWs and community members to situate behaviours in social and economic contexts. These processes challenged entrenched assumptions, broadened understanding of local priorities and fostered more respectful relationships between providers and communities:

*Showing photos--- that led to changes in how nurses in clinics did their work (KI 7)*

These conditions activated mechanisms of recognition and moral engagement. CHWs reported increased respect from clinic staff, while managers described a more grounded appreciation of households’ circumstances. As shared understandings developed, intersectoral engagement around sanitation, water access, waste disposal and substance use became more responsive, with community-generated evidence prompting dialogue with municipal and social-development departments:

*There is now dialogue with municipality officials--- water infrastructure has improved (KI 20, community stakeholder)*
*Communities--- get attention from highly skilled CHWs--- clinics also benefit (KI 17)*

These examples point to a more equity-oriented approach to setting and acting on priorities within routine PHC practice.

***4.4 Institutionalised learning***. By Cycles 4–5, VAPAR had become a regular component of district and sub-district PHC governance. Engagement was consistent and largely system-led, with CHWs, OTLs, OPMs, traditional authorities, clinic committees and sub-district teams participating without external incentives. The CHW peer-learning forum provided ongoing structures for supervision, debriefing, reflective practice and collective problem-solving, and its routines aligned well with existing PHC processes. Formal recognition and written requests from district and provincial authorities to expand VAPAR consolidated institutional legitimacy:

*-- it can be sustained with the leadership of OTLs, working with CHWs who are now trained. They can sustain the process, whatever the topic (KI 17)*
*--- the training of CHWs in Bushbuckridge sub-district--- has resulted in significant and notable improvement in the capacity and performance of this cadre ---the Ehlanzeni health district management team has noted the positive outcomes of the VAPAR programme--- and request the learning and processes from this programme to extended (District DoH letter)*
*--- Reports and feedback from this area demonstrate significant capacity building and the establishment of conducive relationships between local communities, community structures, and health system stakeholders--- we herewith request the VAPAR researchers extend the learning from this programme to other districts in the province (Provincial DoH letter)*

Informants emphasised that sustained institutionalisation depended on supportive leadership, modest resourcing and continued facilitation. Sustainability thus emerged as technically feasible but contingent on organisational commitment and stability:

*Real institutionalisation takes time and is not driven by outsiders but by those in the system doing things in a different way (KI 6)*

Partners began exploring extensions of the participatory approach for outbreak preparedness and emergency obstetric and neonatal care, including in urban settings where real-time, community-linked learning is urgently needed. Overall, mechanisms of trust, credibility and perceived usefulness became embedded in organisational routines, so that the social infrastructure of relationships, reflection and shared practice formed part of system functioning.

***4.5 Transferable processes***. VAPAR generated practices that diffused beyond the original setting because mechanisms were repeatable social processes. As CHWs and supervisors internalised tools for reflective dialogue and peer learning, they adapted them often without external support. Informal diffusion through CHW networks broadened reach, while formal adaptation through SAPRIN and provincial structures extended the model supporting uptake in new settings ^1– 3^ ^60, 61^. Routine planning forums, growing interest in community engagement across SAPRIN nodes, and national momentum for PHC re-engineering were supporting contextual features.

*SAPRIN requires all nodes to have community engagement ---VAPAR may be something that we can introduce --- that additional step happens rarely (KI 14)*

Methodological diffusion also occurred through COMCAT, which extended VA to include social and system circumstances of death. Developed with SAPRIN, COMCAT software and classifications ^24, 25, 62^ have been applied in national mortality studies in South Africa ^63, 64^ and internationally across India, Pakistan, Mozambique, Nepal, Saudi Arabia, DRC ^65– 69^. Team members contributed to WHO VA updates ^4^ and to establishing a WHO Collaborating Centre for Verbal Autopsy at Wits University, strengthening global cause-of-death surveillance.

Conceptual work was also progressed to support institutionalising participation within decentralised health systems. Bossert’s decision-space framework, applied to the MDoH, identified opportunities to expand decentralised authority and create learning spaces to amplify seldom-heard local voices ^56^. Fox’s strategic social accountability concept further informed VAPAR processes, guiding facilitation of ‘state-society synergies’ across communities, the health system, and between peers and non-peers, building local actor power overall ^33^. Finally, the team analysed the process using Popay’s community power-building framework. This helped interpret how collective capacities for joint action emerged through adaptive learning ^58^. These nsights informed a practice framework outlining conditions for co-production and empowerment ^27, 58, 70, 71^.

Academic and capacity-building outputs further extended programme reach. To date, 29 peer-reviewed papers have been published, alongside postgraduate training, participatory-methods workshops in South Africa and Rwanda, and open-source learning materials including CHW manuals, introductory PAR videos and ethics-training modules ^5– 12^. These forms of institutional and epistemic diffusion mark VAPAR’s transition from a discrete research project to an approach shaping routine practice across health systems, research platforms and mortality-surveillance communities.

### 5. Unintended effects and underlying assumptions

No negative consequences were reported, although risks were acknowledged. One concern related to CHW overload, a common challenge in participatory initiatives that expand responsibility without formal support. To avoid raising expectations among unpaid CHWs about employment or remuneration, and following district guidance, Cycle 4 onward focused the training where CHWs were formally contracted. We recognised that this decision could reinforce existing inequities inadvertently between paid and unpaid cadres; however, it reflected an attempt to balance fairness, feasibility, and ethical engagement within prevailing labour arrangements. Positive unintended outcomes were evident: participants described new professional opportunities and social recognition, and replication in contrasting settings demonstrated the model’s adaptability, with similar patterns of rapid trust-building and local ownership. These effects underline the generative nature of participatory learning: once activated, mechanisms of trust, agency and shared purpose tended to produce further capability and connection beyond programme boundaries.

The ToC rested on assumptions of mutual capacity, that community members, CHWs, managers, and researchers would have the basic time, skills, organisational and psychological space to participate meaningfully, alongside minimal discretionary resources, and a stable institutional environment. These conditions were partially met. Commitment and goodwill were strong, but time and funding flexibility remained limited, and social cohesion varied across sites. External shocks such as COVID-19 disrupted continuity but simultaneously validated the model’s adaptability under pressure. Over time, the programme shifted from early optimism about rapid institutional change to a more realistic appreciation of iterative, adaptive learning in open systems. The underlying proposition that co-produced evidence and structured dialogue can strengthen system learning remained valid, but its preconditions proved demanding. Participants emphasised that sustaining learning depends on facilitation, trust, and leadership.

*The system doesn’t change quickly unless there are high-level champions (KI6)*

### 6. Supporting resources

Over ten years, VAPAR operated on a total budget of approximately 1 million GBP. The main funding source was the Joint Health Systems Research Initiative (JHSRI), supported by the MRC, ESRC, Wellcome and the former UK Department for International Development, with additional contributions from the Scottish Funding Council, the Newton/Global Challenges Research Fund, the Medical Research Foundation and the University of Aberdeen. Resources supported the equivalent of approximately 2.5 full-time research posts, with fractional contributions from senior investigators and Wits-based researchers. Relative to inputs, the programme yield was substantial: five action-learning cycles; participation of more than 240 community members; integration with PHC governance structures; 29 academic publications; and broad methodological diffusion. The greatest value lay in the relational infrastructure: trust, communication, and routine spaces for joint reflection, which continues to generate benefits. These findings suggest that durable capability often derives less from material inputs than from strengthened relationships and sustained opportunities for collective sense-making.

## Discussion

This evaluation examined whether, how, why, and in what contexts the VAPAR learning platform generated change in a decentralised district health system. Over a decade, VAPAR created inclusive spaces where communities, CHWs and health officials generated and deliberated over evidence, negotiated meaning, planned action and learned. Across cycles, trust, role clarity, collective sense-making and agency solidified and institutionalised into community health systems learning.

### Embedding learning in context

VAPAR organised participation around recurring encounters between communities, CHWs, and managers. Regular ‘safe spaces’ with careful facilitation interrupted habitual blame and created conditions to enable people to meet and exchange as credible partners. In realist terms, repeated encounters activated latent mechanisms of trust, reciprocity, and shared accountability.

Trust emerged as a key characteristic of the interactions: improving information flows, positioning CHWs as collaborators, and legitimising community knowledge. Trust did not eliminate conflict but made it discussable. This aligns with research on the relational foundations of health system performance ^3, 44^ and extends it by showing that trust within and between communities and health systems can be cultivated through dialogue. As stated by Janz, dialogue is not consensus-building but a mode of difference-holding, a space in which reality becomes thinkable ^72^.

Role clarification was similarly generative. CHWs who had long operated at the margins began to see their work as legitimate, skilled, and of value. Through applying participatory tools in daily work, they developed clearer professional identities and greater confidence in engaging households, peers and managers. Managers, in turn, recalibrated expectations, recognising CHWs as integral members of PHC teams. These reciprocal shifts are consistent with participation scholarship, highlighting that community engagement becomes empowering only when power relations are surfaced and negotiated ^73– 77^. In VAPAR, participation was framed as a shared process of reasoning and agency, with power, position and process continually negotiated and fed back into the process.

Integrating VA data with participatory analysis enabled local actors to connect lived experience with epidemiological evidence. Quantitative data provided entry points for critical and collective reasoning about trends and underlying causes, while photovoice and problem-tree tools powerfully grounded statistics in people’s lived realities. The methodological innovation lay in transforming the information into dialogue, and dialogue into influence, rather than simply combining data types. Evidence then became co-owned, local and relevant.

Similar to other embedded research programmes ^22, 78^, VAPAR demonstrated that co-produced knowledge can support local decision-making embedding evidence processes in practice; supporting change with shared learning. District and provincial managers described VAPAR as the first initiative where research outputs were deliberated locally before action. The Provincial Health Research Committee’s adoption of a research uptake framework and ethics review process drew directly on these experiences, illustrating strengthened governance as well as service delivery.

We observed some limits of embedding participatory learning within bureaucratic systems. The platform gained traction where commitment from district and sub-district managers, as well as clinic operational managers and outreach team leaders, was strong and aligned with programme aims. This was particularly visible at district, sub-district, clinic and community levels. Even though trust and motivation were seen to expand local decision-space, entrenched hierarchies, resource scarcity, and competing institutional pressures constrained it. Participatory mechanisms can generate new learning capabilities but require structural support, relevance and alignment.

Sustainability depended on modest but consistent and supportive leadership and facilitation, both vulnerable to turnover and fluctuations in support. This aligns with evidence that CHW effectiveness hinges on coherent supervision, integration and organisational support ^79– 83^. VAPAR’s peer-learning cycles provided a form of reflective supervision and coordination often missing in routine PHC governance. At system level, the platform performed functions associated with meso-level stewardship: coordinating actors, enabling peer review and strengthening relational accountability ^84, 85^. Embedding in sub-district governance demonstrated that learning can co-evolve when dialogue is institutionalised.

### Community health systems and accountability

VAPAR contributes to a growing literature on critical community health systems that situates equity and accountability within everyday practice ^73, 80, 86, 87^. Through iterative encounters and dialogue, CHWs and community members became credible actors in defining priorities and monitoring services, consistent with contemporary community-led monitoring approaches ^84^. The findings support studies of local committees and civil-society groups showing that participation generates social transformation only when sensitively facilitated, locally grounded, and directly engages power relations ^73, 75, 76^.

In line with calls for improved accountability within and between services and communities ^88^, VAPAR embedded routine relational dialogue into PHC governance and showed how co-produced evidence can support more responsive and adaptive accountability. The process enabled CHWs and community members to raise concerns safely, and negotiate feasible responses through dialogue. Across cycles, shared interpretation of data fostered collective sense-making as a basis for joint action. Few participatory learning platforms have maintained a decade of activity or achieved integration into formal planning processes. Empirically, VAPAR reinforces realist explanations of health system change: once mechanisms of trust, recognition, and collaborative problem-solving are activated within enabling contexts, they can reproduce across cycles and settings.

Theoretically, VAPAR refines Learning Health System models by demonstrating that learning emerges from social infrastructure such as recurring forums, facilitation and trust. Systems-thinking perspectives emphasise human dimensions of relationships, leadership, values and shared meaning as foundational to resilience ^45, 89^. In CR terms, they constitute generative mechanisms through which social structures are reproduced or transformed. Learning thus appears as an emergent property of context-mechanism interactions rather than a managerial tool.

The programme’s diffusion to other provinces and SAPRIN nodes illustrates this point. Expansion occurred not only through formal policy decisions but also through relational learning among researchers and practitioners embedded in government-linked infrastructure. Different sites have re-contextualised mechanisms of dialogue, trust and reflective practice within their own structures and constraints, adapting VAPAR into a heuristic suited to open, changing systems.

Within Mpumalanga, the uptake of the PAR-based CHW training model in the revitalised Health Promotion Programme is particularly significant. Through this integration, VAPAR’s principles of local evidence generation, peer learning and reflection as a management tool have begun to migrate from project to policy, shifting health promotion from routinised information dissemination towards dialogic mutual and relational learning. The experience suggests that iterative, evidence-based participation can be scaled without losing authenticity when the underlying mechanisms of trust, dialogue and reflexivity are preserved, and when the influence of and alignment to social and institutional contexts is explicitly acknowledged and operationally embraced.

### Strengths and limitations

The longitudinal design enabled observation of how mechanisms matured over time and interacted with shifting contexts. The realist and participatory approach enhanced explanatory depth and allowed triangulation across diverse data sources. Coherence between participant accounts, documented actions and institutional uptake strengthens the credibility of the findings. Limitations include the study design and scale, which limited inferences about population-level health outcomes. Researcher involvement in implementation risks biasing interpretation, despite efforts at reflexivity and external advisory oversight. Some mechanisms may have been under-reported where relationships were more fragile or where stakeholders felt constrained in critique. Nonetheless, the analytic focus on context-mechanism-outcome configurations, together with replication of patterns across cycles, supports the explanatory account.

Extending RE with a CR perspective was central to move beyond descriptive accounts and extend explanatory power. Critical realism, with its emphasis on generative mechanisms, layered social realities and the interplay of structure and agency, allowed us to explain how and why VAPAR produced the changes it did, and for whom. It helped connect personal experience of CHWs, community members and managers to professional, organisational and political environments, and to wider PHC reforms. Following Marchal and colleagues, treating mechanisms as dynamic and contingent enabled us to recognise partial expressions of change ^40^, strengthening reasoning about replication. In Bhaskar’s terms, learning emerged as an outcome of dialectical processes in which interactions between people and structures generated new possibilities for action ^90^.

### Implications

Three key implications are highlighted. First, learning infrastructure: regular, facilitated, multi-stakeholder spaces should be recognised as a core function of PHC governance. Such spaces can transform information into capability and sustain motivation where formal incentives are weak. Second, meaningfully and materially strengthening CHWs to work across communities and service domains can humanise rigid and bureaucratic systems. Investment in relational, reflective and facilitative skills has potential to yield significant returns in trust and service quality, complementing and extending technical training. Third, participatory methods can align research, policy and practice. When knowledge is co-produced and deliberated in situ, it gains legitimacy and traction. Future research could further examine how different configurations of facilitation, leadership and decision-space shape the durability and spread of learning platforms.

## Conclusion

Over a decade of iterative practice, VAPAR has shown that even in resource-constrained settings, learning and accountability can be built from the bottom up. The VAPAR platform activated generative mechanisms of trust, role clarity, and collective efficacy within an enabling yet fragile context. This evaluation contributes to the limited empirical literature on learning health systems in LMICs and offers conceptual and practical insights into how participatory learning can be sustained and scaled within decentralised health systems undergoing rapid epidemiological and policy transition.

## Ethics and consent

The study protocols for Cycles 1–3 were approved by the University of Aberdeen (Cycle 1 2017–18 UOA CERB/2019/1/1693, 11/10/2017; Cycles 2–3 2019–21 CERB/2019/1/1693) and the local review board at Wits (Cycle 1 2017–18 M1704115 01/12/2017; Cycles 2–3 2019–21 M1811109, 18/01/2019). An amendment was approved for the final evaluation by the local review board (Evaluation 2022 Amendment to Wits M1811109, 09/09/2022). As part of these processes, research involving human participants must adhere to the Declaration of Helsinki. Written informed consent was gained from all participants in the study. Participants were informed about the nature of the programme and its evaluation, its aims, objectives, procedures and outcomes. Participants were assured that identifying information would be anonymised and would not be disclosed beyond the research team without permission. All participants were free to leave the study at any time and for any reason. Efforts to develop partnerships and processes beyond the programme were sought throughout both the programme and evaluation.

## Declarations

### Data availability

Most of the data supporting this article are contained in the article, its tables and supporting references, and the VAPAR website. The transcripts for the final evaluation interviews contain material which could identify individuals, their views and positions and sharing would therefore violate the confidentiality agreement. Applications can be made by email ( switter@qmu.ac.uk) and would be considered only if transcripts could be heavily redacted to protect identities.
