# Supplementary material for: Community and health systems learning: critical realist evaluation of the VAPAR ‘learning platform’ in rural South Africa 2015-25
Source: Wellcome Open Res. 2026 Apr 6;10:135. Originally published 2025 Mar 18. [Version 3] doi: 10.12688/wellcomeopenres.23381.3 (PMC12775659; doi:10.12688/wellcomeopenres.23381.3)
Supplement: Peer review history

# Reviewer report: Reviewer response for version 2

**DOI:** 10.21956/wellcomeopenres.28148.r142424

**Published:** 2026-02-02

**Authors:** Viola Burau

**Affiliations:** 1 Aarhus University, Aarhus, Denmark

Thank you for this insightful piece of research. 

 It is rare that researchers can follow a programme over a period of ten years, and the longitudinal perspective is a core strength of the paper. The authors draw on rich material from different points in the life cycle of the programme.

 The authors reflect on the evolution of the programme throughout the paper. However, the temporal dimension of the analysis seems to sit uneasily with the realist framing of the evaluation. 

 -The programme has evolved considerably over the past decade. Yet any realist evaluation is based on a programme theory in the singular. The authors refer to the initial programme theory in Figure 1. When was this theory developed? How was the theory refined and when?

 -The material used dates from different points in the life cycle of the programme and associated specific versions of the programme theory. How have the authors tackled the diverging temporalities of the materials used and the (adapted?) programme theory?

 The paper provides thought-provoking insights into the evolution of a programme, but the realist perspective may not be the most fruitful of frameworks. I suggest that the authors

 -throughout the paper reflect more critically about the specific temporalities of the materials and programme theory(ies) used, and

 -expand on the limitations of the realist perspective for longitudinal evaluations, and discuss possible alternative (e.g. Normalization Process Theory).

Is the work clearly and accurately presented and does it cite the current literature?

Yes

If applicable, is the statistical analysis and its interpretation appropriate?

Not applicable

Are all the source data underlying the results available to ensure full reproducibility?

Yes

Is the study design appropriate and is the work technically sound?

Partly

Are the conclusions drawn adequately supported by the results?

Yes

Are sufficient details of methods and analysis provided to allow replication by others?

Yes

Reviewer Expertise:

Comparative health systems and policy, health services research and change

I confirm that I have read this submission and believe that I have an appropriate level of expertise to confirm that it is of an acceptable scientific standard, however I have significant reservations, as outlined above.

# Response

**Published:** 2026-03-11

**Authors:** Sophie Witter

**Affiliations:** Institute for Global Health and Development, Queen Margaret University, Musselburgh, Scotland, UK

We thank Reviewer 5 for this recognition of the longitudinal contribution and for the substantive methodological reflections, which we address below.

 On point 1, We agree that temporality of the programme theory needed clearer specification.   The initial Theory of Change (ToC) in Figure 1 was co-developed during Cycle 1 (2017–19) and published previously. It functioned as the programme’s initial, action-oriented theory at that stage, rather than as a fixed explanatory model for the full 10-year period. At the end of each subsequent cycle, the ToC was revisited through structured reflection with community, CHW, health system and research stakeholders. Illustrative or strategic iterations were also written up and published. Noteworthy refinements included making capacity strengthening explicit, repositioning CHW empowerment and agency as central explanatory mechanisms, and giving greater prominence to institutionalisation as a longer-term outcome. We have now clarified in the Methods how we handled temporalities in the materials. Analysis proceeded in two stages. First, materials were examined within each cycle or programme phase, in relation to the programme configuration and theory current to that period.

 Second, we conducted cross-cycle comparison and retroductive synthesis to identify recurring mechanisms, major adaptations, and emergent longer-term outcomes across the 10-year trajectory. We now make explicit that Table 3 is not presented as the original or singular programme theory for the whole decade, but as a retrospective critical realist explanatory synthesis of the programme’s evolving trajectory. We have also added a limitation acknowledging that the intervention, its theory, and the evaluation frame co-evolved over time, and that any final synthesis may reduce some of the temporal complexity and nuance.

 On point 2, we agree that the manuscript needed to distinguish more clearly between realist evaluation and critical realist evaluation, especially in relation to temporality. Our primary purpose in this paper is not only to ask what worked, for whom, and under what conditions, but to explain how change unfolded over time through interactions between the intervention, its changing contexts, actors’ interpretations, agential responses, and underlying generative mechanisms. We have therefore revised the manuscript to present the study more consistently as a critical realist evaluation of a longitudinally evolving intervention, while clarifying that the initial ToC and subsequent CMO formulations were used as heuristic devices rather than as the sole analytic frame. We have also expanded the limitations to reflect the challenges of applying a retrospective explanatory synthesis to a programme that evolved across multiple cycles. In doing so, we note that alternative or complementary frameworks could have captured dimensions of temporality more directly. In particular, Normalization Process Theory may have further illuminated the embedding and routinisation of participatory practices over time, while an Archer-informed morphogenetic reading may have sharpened the temporal sequencing of structure, agency and change across cycles. We now note this more clearly and explicitly in the Discussion.

# Reviewer report: Reviewer response for version 2

**DOI:** 10.21956/wellcomeopenres.28148.r143450

**Published:** 2026-01-08

**Authors:** Miguel San Sebastian

**Affiliations:** 1 Umeå University, Umea, Sweden

Thanks for allowing me to read this manuscript. I have no major comments. This is a clear and engaging article, and the authors have responded well to the previous, thorough round of revisions.

 I would, however, appreciate clarification on two points. First, the authors may wish to elaborate on the practical advantages of using critical realism beyond realist evaluation. From my reading, the justification for this choice and the insights gained appear similar to those typically achieved through realist evaluation, and further clarification would strengthen the methodological contribution.

 Second, sustainability is discussed in the Results section, but this aspect could be developed further. Expanding on what sustainability means in this context and how it is reflected in the findings would enhance the overall contribution of the paper.

Is the work clearly and accurately presented and does it cite the current literature?

Yes

If applicable, is the statistical analysis and its interpretation appropriate?

Not applicable

Are all the source data underlying the results available to ensure full reproducibility?

Yes

Is the study design appropriate and is the work technically sound?

Yes

Are the conclusions drawn adequately supported by the results?

Yes

Are sufficient details of methods and analysis provided to allow replication by others?

Yes

Reviewer Expertise:

Public health

I confirm that I have read this submission and believe that I have an appropriate level of expertise to confirm that it is of an acceptable scientific standard.

# Response

**Published:** 2026-03-11

**Authors:** Sophie Witter

**Affiliations:** Institute for Global Health and Development, Queen Margaret University, Musselburgh, Scotland, UK

We are grateful to Reviewer 4 for this positive assessment and for the two focused, constructive points below, which we address in turn. On your first point,we have revised the paper to make this distinction clearer. We acknowledge that Realist Evaluation (RE) and Critical Realism (CR) share foundational commitments to generative causality and context-dependence, so the distinction can be quite subtle. We have made some edits to the effect that RE is concerned with what makes a programme work, while CR evaluation is interested in how an intervention works across place and time. This is a different intellectual project from Realistic Evaluation. In our case, the practical advantages of extending to CR arose from three specific analytic needs:  

1. Firstly, CR's concept of morphogenesis (Archer) allowed us to theorise how structures such as CHW statutory roles and functions, supervisory hierarchies, and governance systems/norms were themselves transformed through the programme's iterative cycles. This goes beyond standard RE programme theory.
2. Second, CR's layered ontology (Bhaskar) helped us explain why mechanisms like trust-building operated differently in some contexts (e.g., within DHMT meetings) than in others (e.g., across sectors in Cycle 1), by surfacing the underlying structural conditions that enabled or constrained activation.
3. Third, CR's retroductive logic allowed us to infer latent mechanisms, such as the moral engagement provoked by photovoice, that were not directly observable but were necessary to account for observed patterns.

These distinctions are noted in the Methods (Theoretical and conceptual framing) and Strengths and Limitations.

 On your second point, we agree that sustainability is a central theme that merits greater handling and conceptual clarity. In this paper, sustainability is understood in terms of three key and interconnected levels: Firstly, relational: in terms of the durability of trust, and collaborative relationships and collective agency beyond any single cycle or funded phase. Secondly, institutional: related to the embedding of participatory practices into routine district governance structures, performance reporting, and PHC planning. And third methodological sustainability: as the transfer and adaptation of VAPAR tools and approaches by CHWs, managers, and partner organisations without external facilitation. The analysis demonstrates progress at all three of the levels, though with important caveats: relational capital accumulated over the decade but remained dependent on key champions; institutional embedding and support were achieved in Bushbuckridge and are progressing across Mpumalanga but is not uniform; and methodological transfer was evidenced through the training-of-trainers approach and adoptions through SAPRIN and in PHC in the province via the Health Promotion Programme. This conceptualisation is contained within the Results and Discussion: relational sustainability is addressed in Section 4.4 and the “Embedding learning in context” sub-section of the Discussion; institutional embedding is traced through the programme trajectory and Section 4.4; and methodological transfer is documented in Section 4.5. We do not therefore propose changes to the text on this point, and consider the existing material sufficient to address this point.

# Reviewer report: Reviewer response for version 2

**DOI:** 10.21956/wellcomeopenres.28148.r143449

**Published:** 2026-01-06

**Authors:** Thomas J Foley

**Affiliations:** 1 Newcastle University, Newcastle upon Tyne, England, UK

Thank you for the opportunity to review this interesting paper which correctly identifies a lack of literature on Learning Health Systems in LMICs. I joined the review process at the second stage and therefore did not have sight of the original paper. The authors do however seem to have systematically addressed the comments of earlier reviewers.

 Despite this, the manuscript remains somewhat vague, while at the same time using very dense language. At the risk of reversing course on the advice of reviewer one, it is my view that the reader would benefit from some brief definitions of Verbal Autopsy and Participatory Action Research, how they were linked and what they looked like in this study. I would also like more information to understand the role of CHWs within the health system.

 I didn’t get a clear sense of how the learning cycles and sessions actually ran in practice, who was involved, how often, what format, etc. I would find it impossible to replicate the learning platform from this paper alone. Hopefully, this detail is included in the other 29 papers, but that wasn’t immediately clear.

 Although there is evidence of successful learning cycles with increasingly realistic action planning, it was not clear whether the interventions in the learning cycles were actually successful.

 Even with resource constraints and a lack of digital infrastructure, there is still a role for quantitative measures and it is not clear from this paper whether these are presented elsewhere. Some cost benefit analysis would also be informative given the resource requirement for sustaining the process.

 There are very few negative comments or interpretations in the results and discussion. This is surprising for such a long running project. Learning cycles are often strengthened by reflecting on failures and it would be interesting to see some of that reflected in the paper. At a higher level, it would be interesting to understand where the limitations of this approach lay, in the face of the system constraints described. Coverage of this in the discussion section was superficial.

 Institutionalisation was discussed, but it was unclear whether VAPAR was sustained as business as usual in all sites during the later phase.

 In summary, this is only a partial evaluation of the VAPAR learning platform. Some of the concerns above may be remediable (perhaps by situating this paper within the other 29) and others not, however, given the lack of evidence on this topic in LMICs, it is still very much worth publishing.

 It may be the house style, but it is confusing to see superscripted footnotes alongside references with the same numbering format.

Is the work clearly and accurately presented and does it cite the current literature?

Partly

If applicable, is the statistical analysis and its interpretation appropriate?

Partly

Are all the source data underlying the results available to ensure full reproducibility?

No

Is the study design appropriate and is the work technically sound?

Partly

Are the conclusions drawn adequately supported by the results?

Partly

Are sufficient details of methods and analysis provided to allow replication by others?

No

Reviewer Expertise:

Learning Health Systems

I confirm that I have read this submission and believe that I have an appropriate level of expertise to confirm that it is of an acceptable scientific standard.

# Response

**Published:** 2026-03-11

**Authors:** Sophie Witter

**Affiliations:** Institute for Global Health and Development, Queen Margaret University, Musselburgh, Scotland, UK

We thank Reviewer 3 for this constructive engagement, and for the generous overall assessment. We address each point, below. On your first point, in this revised version, conceptual definitions of Verbal Autopsy, Participatory Action Research, and their linkage in VAPAR are provided in the Introduction (paragraph 4) and Methods (Design and analytical perspective). The role of CHWs is described in the Introduction (paragraph 3), contextualised within South Africa's PHC Re-engineering strategy, Ward-Based Primary Health Care Outreach Teams (WBPHCOTs), and ongoing NHI reforms. We hope these passages, read alongside Table 2 (cycle summary) and Table 3 (CMO configurations), provide sufficient grounding for the reader without re-presenting details contained in the wider body of VAPAR papers, to which readers are referred.  

 On your second point, this is a reasonable observation and reflects space limitations in a single evaluation paper that spans a decade of work. Nevertheless, Table 2 provides a structured summary of all five cycles, including participant numbers, duration, focus, geographic coverage, and key process innovations.  The Methods section summarises and references the published evaluation protocol (Witter et al., 2020, BMJ Open) and the cycle-specific papers, which contain full procedural detail including workshop design, facilitation guides, and participant selection. The training manual, which is available open-access, also provides practical replication guidance.   We have taken care in v2 to make these cross-references explicit so that readers can locate the operational detail without requiring it to be duplicated here. Indeed, replication applicable here pertains to the evaluation design, not to the operational conduct of each learning cycle: the latter is documented in the cycle-specific papers and the open-access training manual, to which the text refers readers explicitly. On your third point, the paper provides evidence of action plan implementation and reported outcomes at several levels: service delivery improvements (Section 4.1), CHW-reported behaviour change (Section 4.2), intersectoral engagement (Section 4.3), and institutionalisation (Section 4.4). We also acknowledge that action plans in early cycles were variably completed; particularly those dependent on resources or higher-level authority, and that this is documented in the cycle-specific papers. The evaluation is intentionally framed as a critical realist, explanatory inquiry into how and why outcomes occurred, rather than a conventional impact evaluation. Within that frame, reported outcomes are triangulated across stakeholder groups and data sources, and the limitations of causal attribution in complex, open systems are acknowledged in the Strengths and Limitations section.   We agree that population-level health outcomes could not be attributed to the programme within the timeframe and design, and this is explicitly noted in Section 4.2. In relation to your fourth point, 1uantitative data are incorporated in the evaluation. VA data processed using InterVA-5 are described in the Methods and discussed in Section 4.2, where mortality trends (including the COVID-19 period) are reported. The earlier finding that over 75% of deaths were linked to at least one community-nominated risk factor is cited in Section 3.2. Programme participation statistics are also reported across cycles (Table 2) and resource inputs are summarised in Section 6 (Supporting Resources), including a total budget of approximately £1 million over ten years and an equivalent of 2.5 full-time research posts. Formal cost-benefit analysis was beyond the scope of this evaluation; however, the resource section provides indicative context on value-for-money. We agree that future work in this area would strengthen the evidence base for learning platforms in LMICs. On your fifth point, We appreciate this comment while noting that the paper does engage critically with tensions, trade-offs and failures, though the reviewer is right that these could be more prominently signalled. Specifically, the paper documents: incomplete action plan implementation in Cycle 1 (Sections 3.3 and 4.1); the disruption and forced redesign caused by COVID-19 (Section 1); the persistent challenges of hierarchical governance, limited operational decision-space, and weak community representation structures (Sections 1 and 5); the risk of CHW overload and the deliberate decision not to train unfunded CHWs (Section 5); the difficulty of sustaining engagement with multi-sectoral partners beyond the initial cycles (Section 3.1); and the concern about whether institutionalisation would be sustained without ongoing facilitation and resource support (Section 4.4). The Discussion (Embedding learning in context; Strengths and Limitations) also addresses structural constraints and reflexivity. This said, we accept that the critical reflections may read as slightly dispersed. On your sixth point, this is an important clarification. VAPAR was not replicated uniformly across all sites; institutionalisation was uneven and contingent. In the Bushbuckridge sub-district, VAPAR became integrated into routine district planning and DHMT structures from 2021 onwards. The CHW peer-learning forum established in 2022 continued beyond the funded cycles. From 2023–25, the approach was adapted and scaled across Mpumalanga's three districts through PHC and the revitalised Health Promotion Programme, and diffused through SAPRIN to other provinces. The paper is explicit that sustainability remained dependent on facilitation, leadership commitment, and modest continued resourcing support, and that these were not uniformly available. The text in Section 4.4 and the final paragraph of the Discussion acknowledges these conditions. On point 7, we are grateful for this overall assessment. The reviewer is right that this paper represents one component of a larger body of work and is explicitly framed as a summative, longitudinal evaluation drawing on and synthesising the wider publication record. We have sought in v2 to make the relationship between this paper and the broader VAPAR corpus clearer, so that readers understand what is and is not claimed here. Finally, on point 8, We agree this is potentially confusing. We have attempted to minimise URL footnotes in v2 and consolidate references to the VAPAR website where possible.

# Reviewer report: Reviewer response for version 1

**DOI:** 10.21956/wellcomeopenres.25776.r133088

**Published:** 2025-09-23

**Authors:** Ana Lorena Ruano

**Affiliations:** 1 University of Bergen, Bergen, Norway

Thank you for the opportunity to review your paper.

 1. The introduction is a little long (and long-winded) in introducing VAPAR. Out of 8 paragraphs, 6 are about VAPAR. I would only include one or two and use this section to better introduce terms and the context. 

 2. There is no need to have introductory paragraphs in a paper. My suggestion is to just convey the information in the section and keep is short and direct. 

 3. Please clarify all data sources in the section and avoid vague phrasing. 

 4. The subheading says data analysis but starts with data collection. Perhaps it shoud be 'data collection and analysis' as they are so closely linked. 

 5. Avoid vague language like ' Quantitative data such as VA were analysed for trends in health indicators for different population groups.' I am not sure what that means. Clearly explain what analysis you did to what data. 

 6. 'Analyzed qualitatively' does not really mean anything. Please develop this section.

 7. The design of the paper is not clear, what methods did you use to evaluate? What type of evaluation? 

 8. I do not undersand the layout of the results/findings because I do not understand what was evaluated and how. I also do not know how the data was analyzed so I cannot speak to more of this paper.

Is the work clearly and accurately presented and does it cite the current literature?

Partly

If applicable, is the statistical analysis and its interpretation appropriate?

I cannot comment. A qualified statistician is required.

Are all the source data underlying the results available to ensure full reproducibility?

No

Is the study design appropriate and is the work technically sound?

No

Are the conclusions drawn adequately supported by the results?

No

Are sufficient details of methods and analysis provided to allow replication by others?

No

Reviewer Expertise:

This reads more like a rather weak report than a paper.

I confirm that I have read this submission and believe that I have an appropriate level of expertise to state that I do not consider it to be of an acceptable scientific standard, for reasons outlined above.

# Response

**Published:** 2025-12-03

**Authors:** Sophie Witter

**Affiliations:** Institute for Global Health and Development, Queen Margaret University, Musselburgh, Scotland, UK

We thank Reviewer 2 for very helpful comments highlighting areas where clarity, structure and methodological detail required improvement. We have addressed every concern directly.

 On comment 1, we have shortened and refocused the Introduction. It now provides a concise health systems and PHC context, defines key concepts (LHS, PAR, realist evaluation) and offers only necessary background on VAPAR. The programme detail has been moved to the Methods and Results.

 On comment 2, all summary paragraphs and preambles have been removed. The paper has been revised throughout for tone that is clearer and more direct.

 On comment 3, we have fully rewritten the Methods clarifying all data sources and their use. The revised version specifies verbal autopsy datasets from the Agincourt HDSS and processed using InterVA5, participatory materials (photovoice, problem trees, action plans), 22 key informant interviews, 9 focus groups, programme documents, provincial health reports, and financial records. Sampling rationale,

 participant characteristics, and data collection languages are now explicitly

 described.

 On comment 4, data collection and data analysis sections have bene revised to reflect full content.

 On 5, in the revised manuscript, we have addressed this in detail. We now

 clearly state that all qualitative data were coded in NVivo using a ToC-derived

 framework, and that identification of CMOs was through cross cycle constant

 comparison. We state use of retroduction to infer underlying mechanisms, and stakeholder validation workshops for credibility. We also specify that VA processed using the InterVA5 probabilistic algorithm.

 On 6, we have expanded the description of the qualitative analysis. All qualitative

 data (interviews, focus groups, and participatory outputs) were imported into NVivo and coded using a framework derived from the co-developed Theory of Change. Inductive sub-themes were added iteratively. Context–mechanism–outcome

 (CMO) configurations were identified through constant comparison across

 datasets and time periods. Reflexive journaling and stakeholder workshops

 were used to validate interpretations. These steps are now fully documented.

 On 7, this is now clearly articulated as a realist and critical realist explanatory evaluation embedded in a participatory action research programme, designed to identify generative mechanisms, contextual contingencies, and emergent system

 change over time and setting. We have added references to Pawson & Tilley and

 Bhaskar to support this framing.

 On 8, The Results have been reorganised into clear subsections, each aligned

 directly to the ToC and realist logic. The Results section has been rewritten

 to highlight how and why outcomes occurred. The Discussion and Conclusion

 have also been rewritten to integrate explanatory insights and demonstrate

 alignment with realist principles. We hope this has strengthened the section

 especially ith clearer signposting between evidence and causal inference. In summary, We have substantially strengthened the technical soundness and transparency of the paper to ensure clarity, reproducibility, and alignment between

 methods, results, and conclusions. All data sources and analytic steps are now

 described in sufficient detail to enable replication. The revised Methods specify use of NVivo 12 for qualitative coding, guided by a co-developed Theory of Change and realist CMO (context/mechanism/outcome) synthesis, while quantitative VA data were analysed using the InterVA5 probabilistic algorithm. Triangulation across datasets and explicit reflexivity procedures enhance analytic rigour. The manuscript now cites current literature on realist evaluation, learning health systems, and participatory research to position the work within the contemporary evidence base. The Introduction has been shortened and refocused, the Methods rewritten, and the Results restructured to highlight explanatory mechanisms consistent with a CR approach. The Discussion and Conclusion have been rewritten to interpret mechanisms of trust, role clarity, and collective efficacy and to show how they underpin uptake of the approach within

 Mpumalanga’s revitalised Health Promotion Programme. Collectively, these revisions address all reviewer concerns and strengthen the conceptual,

 methodological, and practical contributions of the paper.

 We are grateful for both reviewers’ thoughtful critique.

 Their comments led to a substantially improved paper that is clearer, more rigorous, and more

 explicitly realist in reasoning and structure. We believe the revised manuscript now provides a strong explanatory account of how a 10 year participatory learning platform shaped change within a decentralised health system undergoing epidemiological and policy transition. We hope the revised version meets the reviewers’ expectations and the journal’s standards.

# Reviewer report: Reviewer response for version 1

**DOI:** 10.21956/wellcomeopenres.25776.r120951

**Published:** 2025-04-09

**Authors:** Dimitri Renmans

**Affiliations:** 1 Universite Libre de Bruxelles, Brussels, Brussels, Belgium

**Dear authors,**

 Thank you very much for this very interesting paper on the VAPAR learning platform. It gives an in-depth analysis of how a community and health systems learning program managed to increase the uptake of evidence and created strong and respectful relationships between stakeholders. As my main expertise in relation to this paper is the methodology (i.e. a realist-informed study), I will focus on this.

 The study calls itself realist-informed, however, I feel that this term is uncertain and basically empty. What elements of realism ‘informed’ the study? And how? Why is it not fully realist? The reasons why not a full realist study was used are also often not mentioned, as is the case here.

 There may be several reasons why authors call their study realist-informed instead of just realist:

1. Because they are not sure whether the mechanisms are really realist and did not assess the context
2. Because they only used the CMO configuration without truly thinking through the mechanisms
3. Because they did think through the mechanisms, and they are realist, but did not use a CMO configuration or follow all the steps put forward the guidelines on the method and authors are afraid of the ‘realist police’.

Importantly, what makes an evaluation, or a study, realist is not its use of CMO-configurations or the rigorous adherence to the steps of a realist methodology. Instead, what makes a realist study realist is its adherence to the realist way of thinking. This includes the ideas that causality can and should be explained by unearthing the generative mechanisms, the triggering of mechanisms is dependent on the context, different mechanisms may lead to the same outcome and the same mechanisms may lead to different outcomes depending on the context (equifinality and multifinality), and observations by the researchers and the participants are always theory-laden but nonetheless based on an underlying reality.

 Hence, a study is either in line with this way of thinking or not, and hence a study is either realist or not, realist-informed is not one of the options (in my humble opinion). At the same time, it is not important that the researchers have a PhD in philosophy and are experts in realism, but that the study follows the line of reasoning. In this sense many studies not explicitly called realist can still be in line with the realist way of thinking. Regarding the three options above, the first two are not to be called realist (maybe better to call it a theory-based evaluation), while the third option is obviously a realist study, and no such police exists.

 This small excursion to frame the following feedback, because what is the current study under review? On page 5 it mentions that it used a theory of change and followed a realist schema. While it is unclear what is meant with such a schema, I assume that the authors tried to apply the CMO configuration to their theory of change. So let us first look at the theory of change in Figure 1 to see whether this can indeed be seen as realist. When looking at the mechanisms, I think these can certainly be called realist, hence this is already a first indication that we are dealing with an actual realist study. A second important element is that the mechanisms are clearly linked to a context and an outcome, or it is at least discussed how the mechanisms and the context relate to each other and lead to outcomes. Here, the figure is less clear as the identified context is rather general and not linked to the mechanisms. However, since this is just the initial program theory, this can be due to a lack of information and the study may try to dig deeper into this. So, while a realist study would want some more detail and clearer links between context, mechanisms and outcomes, it is acceptable that such specificities are not yet clear at the initial program theory stage. Hence, in that sense, the study should certainly be called ‘realist’.

 However, the theory can use some improvement. First, when creating a theory of change based on a logic model it is important to clearly distinguish between the outputs and the outcomes. Outputs are a direct result of the implementation of the intervention, while outcomes are changes at the level of attitudes, behavior, or knowledge within the beneficiaries of the intervention. This means that the outputs are part of your implementation theory and tell us whether the intervention was correctly implemented, while outcomes are part of your impact theory and tell us whether the intervention had the expected impact on the beneficiaries. This is important because this means that the generative mechanisms should be placed between the outputs and the outcomes and not between the inputs and the outputs as is done right now. While I understand that this theory of change (ToC) was already published in another paper, I believe that it is still useful to update it to make it better.

**Here are some proposed changes:**

**Inputs: **These are activities rather than inputs and lead directly to the outputs.

**Outputs:** ‘Learning platform and shared learning’ can be seen as outputs, however, ‘ownership of locally relevant evidence’ is a mechanism while ‘uptake of locally relevant evidence’ is an outcome. ‘Collective action and learning derived from it’ is either outcome or mechanism.

**Mechanisms: **These are fine and are a clear result of the updated outcomes and may lead in certain circumstances to the described outcomes.

**Outcomes: **Are okay.

**Overall: **Link the mechanisms to specific outcomes and outputs

 Once the initial program theory is developed, it is important that a realist study continues to apply the realist way of reasoning in its analysis. Again, there are no strict rules on how to go about it. The paper shows that it had a participative approach with many different data collection methods and an iterative approach. This fits well with the realist view on data collection which is method neutral and supports the use of triangulation through different methods and different participants.

 What is crucial in a realist analysis is its depth. Again, the study can be seen as in line with the realist approach as it tries to dig deep into the underlying mechanisms that lead to the observed outcomes. However, the way these are structured in the results section does not align with the realist way of thinking about causality. The paper discusses the context, mechanisms, underlying assumptions (which look like contextual elements), and outcomes separately; however, it is crucial in a realist analysis to make the link between these different elements. A realist study tries to understand which elements in the context triggered which mechanisms leading to which outcomes. It’s this understanding of how certain conditions were important for triggering (for example, the trust mechanism) and leading to improved engagement that can help us to improve this and other interventions by making sure that these contextual conditions are present and/or are strengthened.

 It is because of this that this study cannot be called a realist study and, arguably, not a realist-informed study either. This crucial element can however be integrated by restructuring the text and making the links which I am sure you know very well. There are different ways in which you can structure the result section. It can be presented in a chronological way stressing the importance of the impact of the earlier phases on the latter phases. It can be structured by focusing on certain outcomes stressing how different mechanisms and contextual conditions interact to lead to certain outcomes, or it can be structures according to certain domains of the overall program theory, for example, one part on collaboration, one on relationships, and one on individual competencies/motivation. A final way to structure the results section in a realist way is according to stakeholders, without of course neglecting the important interactions between them.

 So, overall, this is a great study with great depth and interesting findings; however, the way it is presented and analyzed is more in line with a theory-based evaluation than a realist evaluation. However, all the elements to transform this into a realist evaluation are present and I belief that when analyzed and structured in one of the proposed ways this may add even more depth and understanding to the study. However, this is a choice to be made by the authors and the study is still valuable as it stands. However, personally, I would advice against the use of the term ‘realist-informed’.

Is the work clearly and accurately presented and does it cite the current literature?

Yes

If applicable, is the statistical analysis and its interpretation appropriate?

Not applicable

Are all the source data underlying the results available to ensure full reproducibility?

Yes

Is the study design appropriate and is the work technically sound?

Partly

Are the conclusions drawn adequately supported by the results?

Yes

Are sufficient details of methods and analysis provided to allow replication by others?

Yes

Reviewer Expertise:

Realist and theory-based evaluation, Health systems research

I confirm that I have read this submission and believe that I have an appropriate level of expertise to confirm that it is of an acceptable scientific standard.

# Response

**Published:** 2025-12-03

**Authors:** Sophie Witter

**Affiliations:** Institute for Global Health and Development, Queen Margaret University, Musselburgh, Scotland, UK

We thank Reviewer 1 for an exceptionally thoughtful review that sharpened our conceptual and methodological reasoning. The clarity offered on realist philosophy and realist evaluation was invaluable. We have made substantial revisions throughout the manuscript, fully addressing each point.

 On comment 1, we agree the term realist-informed is vague and risks signalling that the study is not fully realist. The reviewer requests clarifying whether and how realist principles were applied, and why the approach was not framed as a full realist evaluation from the outset. We agree entirely that the previous terminology was imprecise. In the revised manuscript, we remove all use of “realist-informed.” We explicitly describe the design as a realist and critical-realist explanatory evaluation embedded within a participatory action-research (PAR) programme. We also clarify why the evaluation did not follow all procedural stages of a formal realist evaluation because VAPAR began as a PAR programme (2015) and the realist evaluation was integrated as the programme matured, the evaluation was necessarily embedded and iterative rather than initiated de novo using RE protocols.   In the revised manuscript, we also explicitly articulate which realist principles shaped the design and analysis, including: generative causation, context-dependent mechanism activation, theory-laden observation, and open-system emergence across cycles. These clarifications are made in the Methods (Design and analytical perspective) and reinforced in the Discussion. As part of foundational theoretical and structural revisions, we have extended the evaluation window in order to trace the origins, maturing, and institutionalisation of the model. The earlier pilot phase (2015–16) was essential for understanding the development of COMCAT and the programme’s epistemic foundations; the later uptake phase (2023–25) was crucial for explaining how and why mechanisms diffused, scaled, and became embedded within the provincial Health Promotion Programme and through SAPRIN. The explanatory account of institutionalisation and mechanism reproduction is now coherent and complete with findings are presented as context-mechanism-outcome (CMO) patterns across this ten-year trajectory.

 On comment 2, we appreciate the concern that in realist logic, mechanisms should sit between outputs and outcomes, not between inputs and outputs. The ToC did not clearly differentiate implementation theory from impact theory. In response, we have fundamentally revised the ToC structure and narrative. Inputs now refer only to programme activities and enabling resources. Outputs now refer to direct and observable products of these activities. Mechanisms have been positioned to reflect their generative roles. Outcomes include behavioural,relational, organisational and governance changes and explicit links are made between each mechanism and its associated outcomes. This has substantially strengthened the internal logic of the evaluation and aligns directly with the reviewer’s guidance.

 On comment 3, we fully agree with the comment that realist analysis requires explicit CMO reasoning: how specific contextual conditions trigger specific mechanisms

 to produce specific outcomes. In the revised paper, the entire Results section has been restructured to foreground CMO patterns. Each domain now presents relevant contextual features, how those features triggered or constrained mechanisms, and the outcomes produced. These narrative CMOs make the causal reasoning transparent and firmly anchor the findings in realist logic. We have also distilled out specific CMO configurations and present them in Table 3.

 On comment 4, We acknowledge that the study contains all the elements of realist evaluation but was not previously presented in a realist structure. We have adopted the reviewer’s advice and restructured the Results using ToC domains integrated with chronological emergence, which maintains coherence across five cycles and the uptake phase, and highlights how mechanisms matured over time. The revisions align naturally with realist explanatory reasoning This restructuring substantially strengthens the analytic coherence of the manuscript and is a significant change to the paper.
